# Mapping the “Supply–Demand–Flow” of Ecosystem Services for Ecosystem Management in China

**DOI:** 10.1002/advs.202522070

**Published:** 2026-04-15

**Authors:** Yikun Zhang, Yadong Yang, Yongsheng Wang, Guirui Yu

**Affiliations:** ^1^ State Key Laboratory of Efficient Utilization of Arable Land in China Institute of Agricultural Resources and Regional Planning Chinese Academy of Agricultural Sciences Beijing China; ^2^ School of Plant and Environmental Science Virginia Tech Blacksburg Virginia USA; ^3^ Institute of Geographic Sciences and Natural Resources Research Chinese Academy of Sciences Beijing China

**Keywords:** china, demand, ecosystem management zoning, ecosystem service, flow, supply

## Abstract

Ecosystem services (ES) link ecosystems and human societies, yet approaches to ecosystem service flow (ESF) are constrained by ambiguous definitions and limited process‐oriented and scale‐sensitive classifications. Here, we developed a “supply–demand–flow” framework distinguishing potential and actual supply and demand, and classifying ESF into in situ, interior, and exterior flows. Integrating multi‐source datasets, biophysical models (InVEST, RUSLE, i‐Tree), and socio‐economic accounting based on population‐consumption dynamics and sectoral statistics, we mapped nine ES across China from 2000 to 2020 at 1 km resolution. Results revealed pronounced ES supply and demand spatio‐temporal heterogeneity. While the national ESF pattern remained stable, most services showed increasing exterior reliance, whereas water yield and tourism recreation became more locally sustained. Using flow balance and demand fulfillment as policy‐proximal indicators, five management zones were delineated: local‐sustained counties dominated southern China (∼30%); local‐pressured types occurred in the Northeast, Qinghai–Tibet, and southwestern areas (∼15%); external‐pressured types occurred in arid northwestern regions (1%–3%); southeastern coasts shifted toward external‐sustained types (∼30%); and dynamic‐transitional counties (∼20%) were scattered nationwide. By decomposing supply‐demand relations into supply realization, spatial reallocation, and demand fulfillment, our framework helps align ecological functions with management priorities and offers insights for reconciling development and conservation in comparable socio‐ecological contexts.

## Graphical abstract

This study develops a “supply–demand–flow” framework clarifies how ecosystem services move between regions by distinguishing potential and actual supply and demand. Using integrated biophysical–socioeconomic modeling, nine services in China were mapped. Results identify growing external reliance, local persistence of water yield and tourism, and five management zones guiding balanced ecological development.

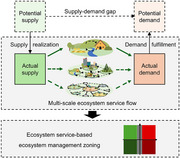

## 1 Introduction

Achieving global sustainable development and human well‐being fundamentally depends on the capacity of ecosystems to provide essential goods and services [1, 2]. These benefits, commonly conceptualized as ecosystem services (ES), serve as vital connections between ecosystems and social systems [3, 4]. Since mismatches between ecological processes and human needs persistently undermine long‐term sustainability, clarifying the relationships among different dimensions of ES is therefore crucial [5]. In particular, for macro‐scale ecosystem management, understanding how ES are generated, allocated, and aligned with societal needs provides the foundation for evidence‐based planning and decision‐making [6, 7].

ES supply and demand represent the most immediate and observable linkage between ecosystems and human societies [8, 9]. Plenty of research has focused on quantifying supply‐demand relationship at global, national, regional, and basin scales. For example, existing studies analyzed spatial mismatches between ES supply and demand, assessed ES budgets, or examined social–ecological networks to support ecological planning and policy design [10, 11, 12, 13, 14]. Beyond the ES supply‐demand relationship, ecosystem service flow (ESF), the processes of services transferring from ecosystems to human societies, have become increasingly emphasized [15, 16]. Currently, researchers have explored ESF from multiple perspectives. Some studies classify ESF according to transmission distances, directional pathways, or transfer forms, thereby distinguishing different ESF types across space [15, 16, 17, 18]. Other studies focus on quantifying ESF by analyzing supply–demand relationships or by developing modelling approaches to simulate specific service transfer processes [5, 7, 19, 20, 21]. In addition, ESF concepts have increasingly been incorporated into ecosystem management and policy instruments, such as payment for ecosystem services (PES), carbon markets, and ecological compensation schemes, to better link ecosystem service providers with beneficiaries [20, 22, 23].

Nevertheless, existing studies on ESF remain unsettled in several respects. First, the definition of ESF remains ambiguous, and existing research has primarily concentrated on depicting flow pathways or measuring flow volumes [18, 20, 24]. However, the fundamental source (supply) and endpoint (demand) of these flows have often not been clearly defined—particularly in distinguishing between potential and actual supply, or potential and actual demand—thereby reducing the applicability of current typologies for ecosystem management. Second, most ESF studies tend to examine limited ecosystem functions or rely on static simulations that treat ESF as time‐invariant snapshots, without explicitly accounting for temporal variations or feedbacks between ecosystem supply and socio‐economic demand [25, 26]. These limitations highlight the necessity of developing a more process‐oriented and scale‐sensitive classification system, which can better capture the spatial heterogeneity of ESF and address the shortcomings of existing distance‐based typologies.

As one of the most geographically vast and socioeconomically diverse countries globally, China faces pronounced imbalances between ecological conditions and development levels across regions [27]. Rapid urbanization and agricultural modernization have generated typical and representative patterns of ES supply, demand, and flow, while simultaneously intensifying ecological challenges such as water scarcity, land degradation, and biodiversity loss [27, 28, 29]. Ecosystem management in China is commonly organized at the county level. Counties often pursue distinct functional priorities, including food production in agricultural regions, strict conservation key ecological function counties, and balancing development and ecological security in rapidly urbanizing regions [30, 31, 32]. This diversity highlights the heterogeneity of ecosystem management goals across scales. While natural units such as servicesheds represent the ideal boundaries for capturing ecological flow processes, they often lack a unified governing body [33, 34]. In this context, using counties as the primary analytical unit allows the “supply–demand–flow” diagnostics to remain policy‐proximal and actionable within existing governance frameworks. Since ESF across and within county boundaries plays a critical role in shaping ecosystem management outcomes, China represents a highly suitable and policy‐relevant context for developing and testing a scale‐oriented ESF classification framework.

Therefore, this study aims to establish a spatially explicit classification framework of ESF grounded in the integrated “supply–demand–flow” perspective. The specific objectives are (1) to clarify the conceptual definitions of ES supply, demand, and flow, and on this basis, to develop a multi‐scale approach for ESF measurement; (2) to reveal the spatio‐temporal dynamics of China's ES supply, demand, and flow during 2000–2020 at a fine spatial resolution (1 km × 1 km); and (3) to delineate county‐scale ecosystem management zones in China according to ES characteristics. Our contributions are threefold: (1) Conceptually, we refined the existing supply–demand framework by distinguishing potential from actual supply and demand, and by introducing a distance‐sensitive classification of service flows. We also proposed a conceptual process chain that links supply realization, spatial reallocation, and demand fulfillment as a heuristic for diagnosing mismatches. (2) Methodologically, we conducted a 20‐year nationwide 1‐km assessment of nine key services that integrate ecological and socioeconomic datasets to represent supply, demand, and flows. (3) Practically, we transformed the “supply–demand–flow” dynamics into two policy‐proximal indicators (the flow balance index and the demand fulfillment ratio) and a five‐zone management scheme to support differentiated interventions. These indicators reflect the balance between local service provision and external transfers as well as the extent to which local demand is fulfilled, thereby providing actionable signals for ecosystem management. Together, these conceptual, methodological, and practical contributions advance the understanding of ESF and provide a framework that can inform ecosystem management and policy design in China and potentially in other regions with comparable socio‐ecological contexts.

## 2 Theoretical Framework

**FIGURE 1:**
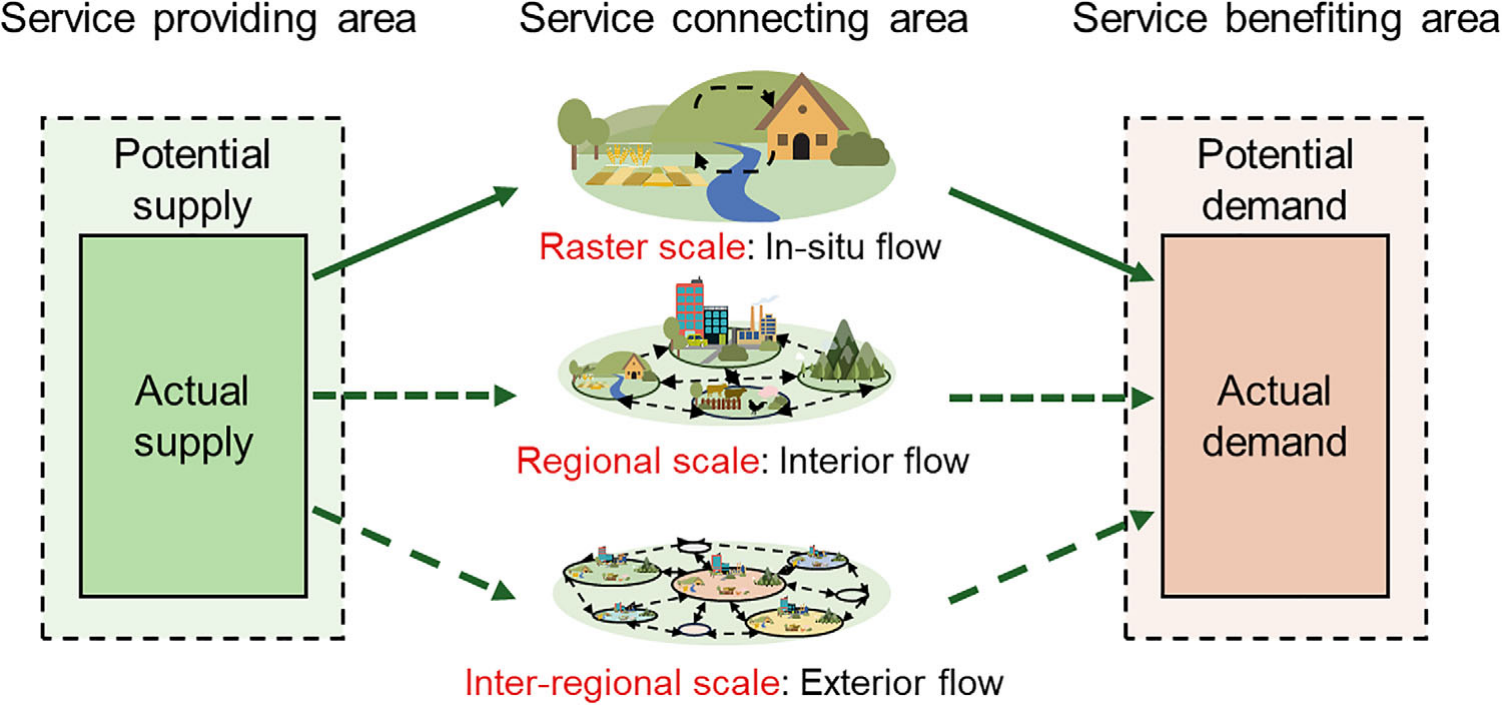
Theoretical framework of Ecosystem supply, demand, and flow.

Ecosystem service supply is the source of ES processing, emphasizing the ability of ecosystems to produce products and services for humans through their structure and function [8]. Existing studies mainly divide ES supply into potential supply and actual supply. Potential supply refers to the maximum level of ESs that an ecosystem could theoretically provide under a specific spatio‐temporal scale, considering its biophysical and socio‐economic characteristics. Actual supply refers to the quantity of ESs that are effectively delivered at a specific spatio‐temporal scale [9, 35, 36] (Figure 1).

Ecosystem service demand is the endpoint of ES processing, emphasizing the part of services that generate utility to meet the developmental needs of humans [8]. Similarly, it includes potential demand and actual demand. Potential demand refers to the degree of expectation and preference for ESs at a certain spatio‐temporal scale under the existing allocation of scarce resources, while actual demand refers to the amount of ESs that are effectively received and utilized by beneficiaries at a specific spatio‐temporal scale [9, 35, 36] (Figure 1).

Ecosystem service flow (ESF) is the linkage between ES supply and demand [16, 37]. However, existing research has no standardized definition of this concept [6, 24, 38, 39]. In this study, ESF is defined as the spatial transfer process through which ecosystem services move from supply areas to beneficiaries, linking ecosystem service supply with human demand. Within this framework, potential supply (PS) and potential demand (PD) describe the biophysical and socio‐economic bounds within which ESF may occur, whereas the ESF components represent the realized transfers of ecosystem services after spatial coupling between actual supply (AS) and actual demand (AD) (Figure 1).

With the co‐influence of natural and socio‐economic factors, ESs’ supply and demand usually exhibit spatial heterogeneity, resulting in mismatches between service supply and demand. Consequently, ESF often occurs across space, linking supply and demand regions. Previous studies have proposed various classifications to better characterize the spatial flows of ESF. For instance, Costanza [38] categorized ESF into five types: global non‐proximal, local proximal, directional flow‐related, in situ, and user movement‐related. Fisher, et al. [39] proposed a typology consisting of in situ flow, omni‐directional flow, and directional flow. Wang, et al. [16] classified ESF into two categories: intra‐regional and inter‐regional flows. Building on these existing frameworks, this study develops a classification based on the spatial distance between service supply and demand, emphasizing the multi‐scale nature of ESF and regional management. Specifically, ESFs are categorized into three types: in situ flow (ISF), interior flow (IF), and exterior flow (EF) (Figure 1).

In practice, spatial distance is represented through a hierarchical partition of grids and administrative units. ISF refers to ultra‐local benefits occurring within grid cells (1 km × 1 km), IF refers to flows within a county, and EF refers to flows that cross county boundaries. Although this partition‐based classification does not measure continuous geographic distance directly, it provides a practical proxy for increasing spatial separation between service supply and demand while maintaining consistency with administrative units commonly used in ecosystem management (Figure 1). Consequently, depending on travel distance, the same ES may belong to different flow types. For example, food produced for local rural consumption is classified as ISF, food transported within a county as IF, and food transported across counties as EF. This classification highlights the multi‐scale nature of ESF, supporting spatially explicit ecosystem management at different scales.

## 3 Results

### 3.1 ES Potential Supply and Demand

**FIGURE 2:**
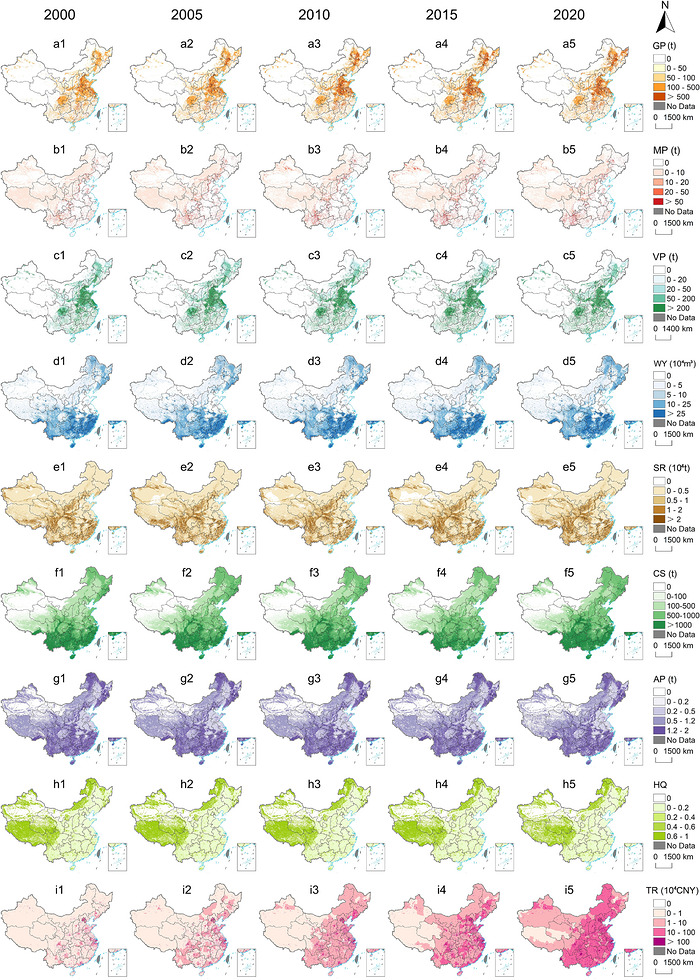
ES potential supply in China. Panels a–i represent nine ecosystem services: (a) grain production (GP), (b) meat production (MP), (c) vegetable production (VP), (d) water yield (WY), (e) soil retention (SR), (f) carbon sequestration (CS), (g) air purification (AP), (h) habitat quality (HQ), and (i) tourism recreation (TR). Subpanels 1–5 correspond to the years 2000, 2005, 2010, 2015, and 2020, respectively (e.g., a1–a5 for GP in 2000, 2005, …, 2020). Results are original physical values presented at a 1 km × 1 km resolution (n ≈ 9.5 × 10^6^ pixels). Data represent the absolute quantified state of the entire study area; thus, frequentist *p* values and significance testing are not applicable to this mapping approach.

Potential ES supply in China exhibited evident spatial heterogeneity, closely linked to the natural environment and resource endowment. Except for a substantial increase in tourism recreation supply, the potential supply capacities of other ES remained relatively stable during the study period (2000–2020) (Figure 2). Specifically, grain production exhibited an increasing trend from 513 million tons to 693 million tons, with a higher production in the southeast and lower in the northwest (Figure 2a). Main production areas included the North China Plain, Northeast China Plain, and Sichuan Basin, with notable increases in eastern and southern Heilongjiang and northern Xinjiang. Meat production increased from 57 million tons to 84 million tons, with intensive feeding concentrated in the southeast and extensive pastoral farming in the northwest (Figure 2b). High‐supply areas include the Agro‐pastoral ecotone in northern China and southwest provinces like Yunnan and Guizhou. Vegetable production increased from 475 million tons to 780 million tons, spatially similar to grain production, concentrated in the North China Plain, Middle‐Lower Yangtze Plain, and Sichuan Basin (Figure 2c). Water yield remained stable, increasing from 681.1 to 759.3 billion m^3^, mainly distributed in mountainous areas south of the Yangtze River (e.g., Wuyi Mountains, Nanling, Wuling Mountains), the Changbai Mountains, and Greater and Lesser Khingan Ranges in the northeast (Figure 2d). Soil retention rose slightly from 37.69 to 39.90 billion tons, showing high values primarily in the Qinghai‐Tibet Plateau, Loess Plateau, and Qinba Mountains (Figure 2e). Carbon sequestration increased from 4.52 to 5.30 billion tons, prominently in forest‐rich regions such as the Yunnan‐Guizhou Plateau and Changbai Mountains, while desertification regions in northwest China recorded the lowest supply (Figure 2f). Air purification slightly declined from 81.27 to 78.35 billion tons, with stronger capacity in southern regions and northeastern forested areas (Figure 2g). Habitat quality was relatively stable, with high‐quality areas (>0.5) primarily in the Qinghai‐Tibet Plateau and mountainous regions, and low‐quality areas (≤0.25) in desertified northwest regions (Figure 2h). Tourism recreation supply sharply increased from 897.6 billion Chinese yuan (CNY) to 23,048.3 billion CNY, evolving from scattered to continuous spatial distribution in the southeastern areas (Figure 2i).

**FIGURE 3:**
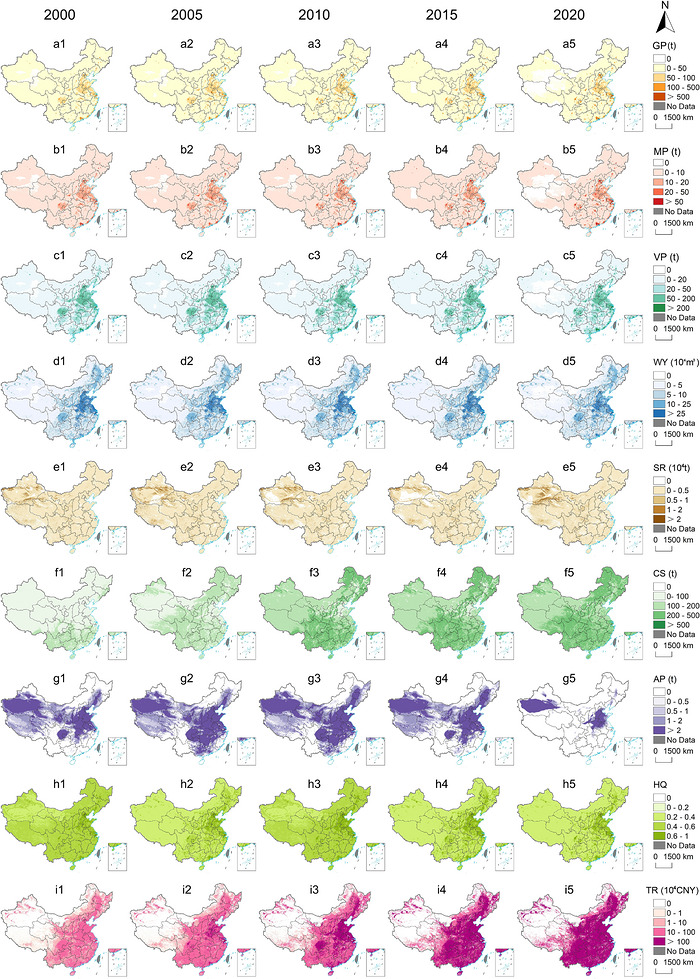
ES potential demand in China. Panels a–i represent nine ecosystem services: (a) grain production (GP), (b) meat production (MP), (c) vegetable production (VP), (d) water yield (WY), (e) soil retention (SR), (f) carbon sequestration (CS), (g) air purification (AP), (h) habitat quality (HQ), and (i) tourism recreation (TR). Subpanels 1–5 correspond to the years 2000, 2005, 2010, 2015, and 2020, respectively (e.g., a1–a5 for GP in 2000, 2005, …, 2020). Results are original physical values presented at a 1 km × 1 km resolution (n ≈ 9.5 × 10^6^ pixels). Data represent the absolute quantified state of the entire study area; thus, frequentist *p* values and significance testing are not applicable to this mapping approach.

Potential ES demand was closely associated with human activities (Figure 3). Potential grain demand increased from 126 to 140 million tons, concentrated in densely populated urban clusters, particularly in southeastern China (Figure 3a). Meat demand rose from 27.33 to 30.33 million tons, predominantly in urbanized regions such as the North China Plain, Yangtze River Delta, Chengdu‐Chongqing, Middle Yangtze, and the Guangdong‐Hong Kong‐Macao Greater Bay Area (Figure 3b). Vegetable demand increased similarly from 137 to 152 million tons, with broader and higher spatial demand concentrated in urbanized regions compared to meat (Figure 3c). Water yield demand increased from 539.8 to 573.7 billion m^3^, primarily in densely populated and agriculturally productive areas like the North China Plain, Northeast China Plain, and rapidly urbanizing areas such as the Chengdu‐Chongqing region (Figure 3d). Soil retention demand grew notably from 19.50 to 36.69 billion tons, particularly in desertified and erosion‐prone areas in northwest China (Figure 3e). Carbon sequestration demand significantly increased from 664 to 2.14 billion tons, mainly in southwest and northeast regions responding to rising carbon emissions (Figure 3f). Air purification demand decreased from 14.43 to 8.36 million tons, predominantly concentrated in the North China Plain and southern Xinjiang, with an initial expansion (2000–2010) followed by contraction (2010–2020) (Figure 3g). Habitat quality demand remained stable at approximately 0.44 per unit area, higher in economically active regions such as the North China Plain and Yangtze River Delta (Figure 3h). Tourism recreation demand sharply increased from 897.6 billion CNY to 23,048.3 billion CNY, expanding from initially scattered urban centers to extensive southeastern regions (Figure 3i).

### 3.2 ES Actual Supply and Demand

**FIGURE 4:**
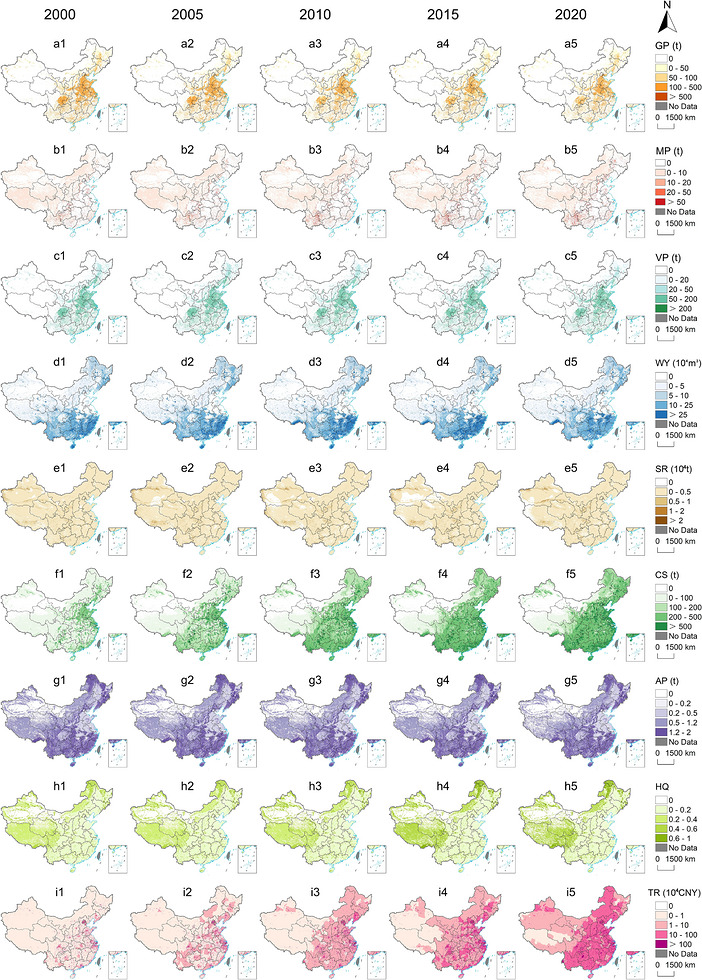
ES actual supply in China. Panels a–i represent nine ecosystem services: (a) grain production (GP), (b) meat production (MP), (c) vegetable production (VP), (d) water yield (WY), (e) soil retention (SR), (f) carbon sequestration (CS), (g) air purification (AP), (h) habitat quality (HQ), and (i) tourism recreation (TR). Subpanels 1–5 correspond to the years 2000, 2005, 2010, 2015, and 2020, respectively (e.g., a1–a5 for GP in 2000, 2005, …, 2020). Results are original physical values presented at a 1 km × 1 km resolution (n ≈ 9.5 × 10^6^ pixels). Data represent the absolute quantified state of the entire study area; thus, frequentist *p* values and significance testing are not applicable to this mapping approach.

The actual supply (AS) of ES in China exhibited similar spatial patterns to the potential supply (PS) but with lower quantities and dynamic temporal changes (Figure 4). Specifically, grain AS decreased from 252 to 189 million tons (2000–2020), and the ratio of PS converted to AS declined from 49.1% to 27.3%, mainly distributed in the North China Plain and Sichuan Basin (Figure 4a). Meat AS increased markedly from 28 to 53 million tons, with PS‐to‐AS ratio rising from 49.1% to 63.1%, showing an evenly distributed spatial pattern nationwide and slightly higher values in southwestern regions (Figure 4b). Vegetable AS decreased from 144 to 132 million tons, and the PS‐to‐AS ratio dropped from 30.3% to 16.9%, primarily in the North China Plain and Sichuan Basin (Figure 4c). Water yield AS increased slightly from 5.43 to 5.54 trillion m^3^, fluctuating at a PS‐to‐AS ratio of 70%–80%, and remained spatially higher in southern regions (Figure 4d). Soil retention AS rose from 8.22 to 8.74 billion tons, maintaining an approximate PS‐to‐AS ratio of 22%, spatially uniform nationwide with slightly higher values in the northwest (Figure 4e). Carbon sequestration AS notably increased from 615 to 2080 million tons, with PS‐to‐AS ratio increasing from 13.6% to 39.2%, prominently in southwest and eastern coastal areas (Figure 4f). Air purification AS decreased slightly from 8.18 to 7.89 million tons, with nearly all PS converted to AS, higher in southern and northeastern forested regions (Figure 4g). Habitat quality and tourism recreation were treated as state‐based services in the ESF accounting framework (Section 6.1 and Table S1); therefore, AS equals PS, resulting in identical spatial distributions (Figure 4h,i).

**FIGURE 5:**
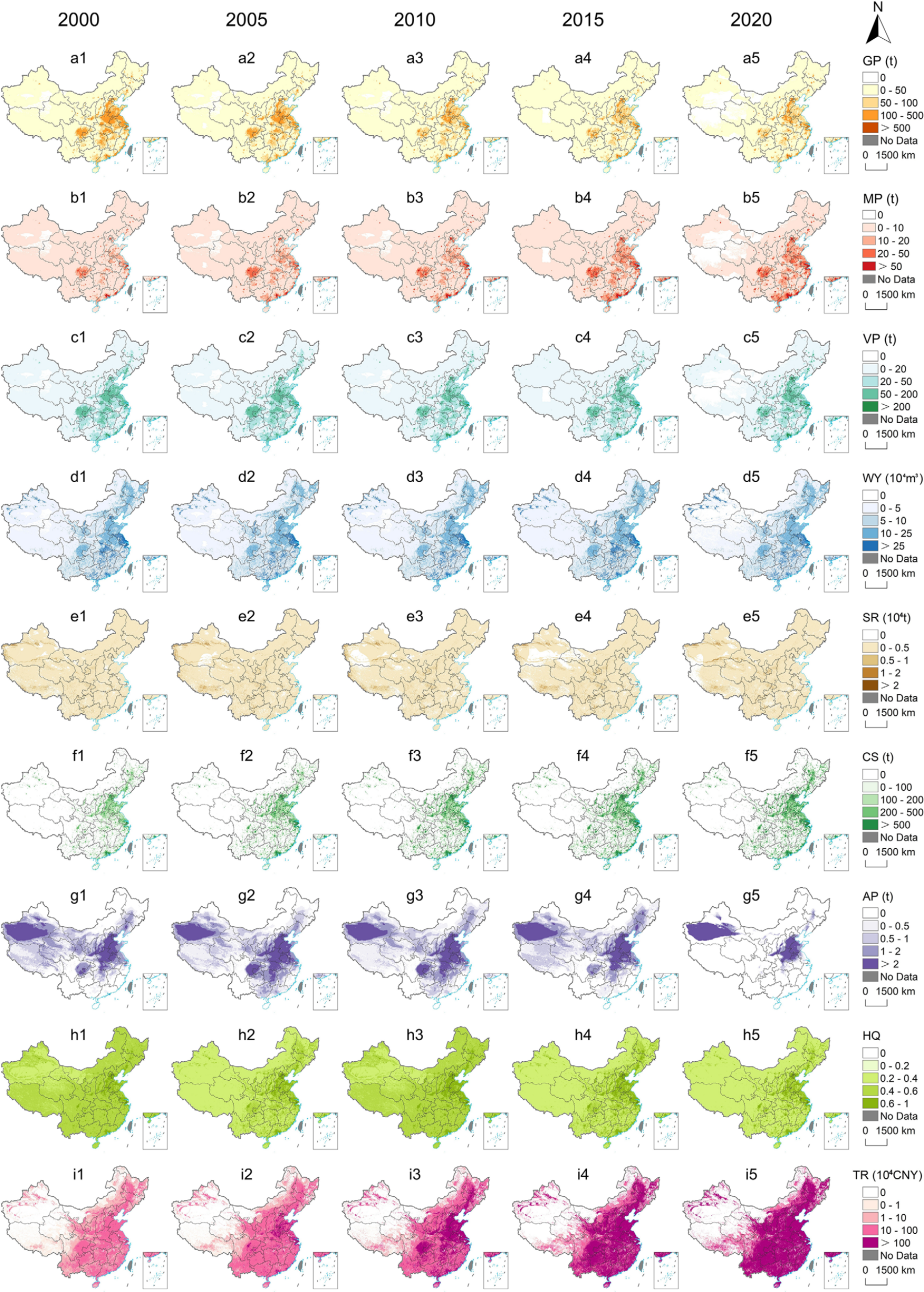
ES actual demand in China. Panels a–i represent nine ecosystem services: (a) grain production (GP), (b) meat production (MP), (c) vegetable production (VP), (d) water yield (WY), (e) soil retention (SR), (f) carbon sequestration (CS), (g) air purification (AP), (h) habitat quality (HQ), and (i) tourism recreation (TR). Subpanels 1–5 correspond to the years 2000, 2005, 2010, 2015, and 2020, respectively (e.g., a1–a5 for GP in 2000, 2005, …, 2020). Results are original physical values presented at a 1 km × 1 km resolution (n ≈ 9.5 × 10^6^ pixels). Data represent the absolute quantified state of the entire study area; thus, frequentist *p* values and significance testing are not applicable to this mapping approach.

The actual demand (AD) of ES equaled the AS in total quantity according to the algorithm (Section 6.1), and exhibited distinct spatial patterns (Figure 5). Specifically, grain AD generally met the potential demand (PD), spatially showed higher values in southeastern regions (e.g., Middle‐Lower Yangtze, North China Plain), but gradually declined during the study period (Figure 5a). Meat AD, meeting the PD, concentrated in major urban clusters including the Yangtze River Delta, Chengdu‐Chongqing, and Guangdong‐Hong Kong‐Macao Greater Bay Area, with rapid increases in southwest, southeast, and northern China (Figure 5b). Vegetable AD slightly decreased, resulting in minor shortfalls compared to the PD (nationally recommended per‐capita vegetable requirement), mainly concentrated in major urban regions similar to meat (Figure 5c). Water yield AD largely met PD, spatially higher in eastern and northern regions. Notable increases occurred in major agricultural and urbanization zones (e.g., North China Plain, Northeast China Plain, Chengdu‐Chongqing area, and northern Xinjiang) (Figure 5d). Soil retention AD exhibited an increasing shortfall compared to PD, with highest demands in desertified areas (e.g., southern Xinjiang, northern Tibet, western Inner Mongolia, Qinghai) and lower values in central‐eastern China (Figure 5e). Carbon sequestration AD generally met PD, higher in eastern coastal regions, and increased significantly in energy‐development regions (e.g., Hohhot‐Baotou‐Ordos‐Yulin) (Figure 5f). Air purification AD initially showed a substantial deficit compared to PD, which was largely alleviated by 2020, with highest demands persistently in northwestern China (Figure 5g). Habitat quality AD remained stable and consistent with PD, spatially higher in both northwestern and southeastern regions (Figure 5h). Tourism recreation AD increased markedly, expanding from urban clusters (North China Plain, Sichuan Basin, Northeast China Plain) toward extensive southeastern regions (Figure 5i).

### 3.3 Ecosystem Service Flow

**FIGURE 6:**
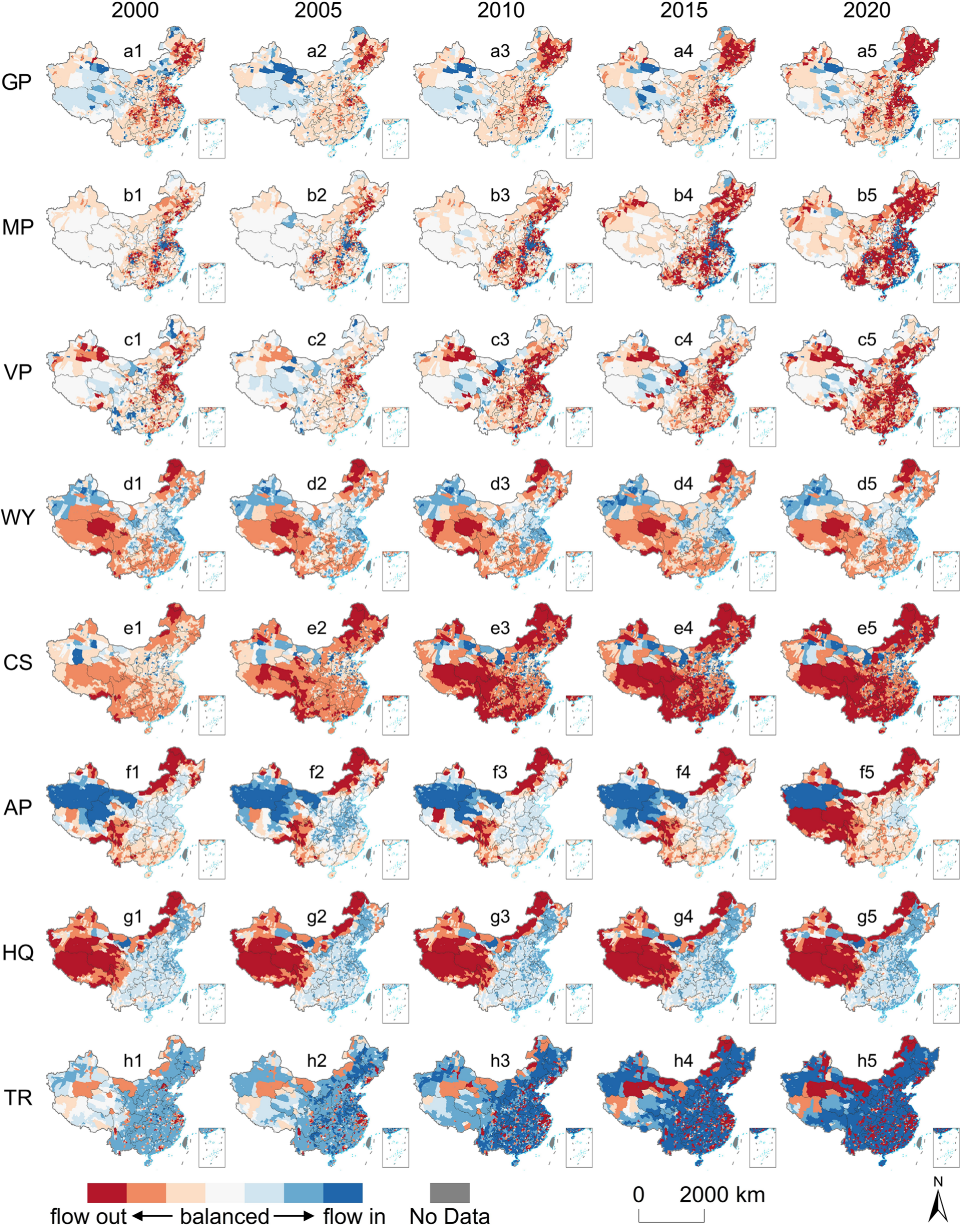
Overall spatial dynamics of ESF in China. Panels a–h represent eight ecosystem services included in ESF assessment: (a) grain production (GP), (b) meat production (MP), (c) vegetable production (VP), (d) water yield (WY), (e) carbon sequestration (CS), (f) air purification (AP), (g) habitat quality (HQ), and (h) tourism recreation (TR). Subpanels 1–5 correspond to the years 2000, 2005, 2010, 2015, and 2020, respectively (e.g., a1–a5 for GP in 2000, 2005, …, 2020). Red indicates net flow out, blue indicates net flow in, and light colors indicate more balanced conditions. Results are aggregated and presented at the county level (n = 2846 administrative units). Data represent the absolute quantified state of the entire study area; thus, frequentist *p* values and significance testing are not applicable to this mapping approach.

The overall ESF in China exhibited distinct spatial heterogeneity (Figure 6). Grain products mainly flowed from the agricultural plains to the southeastern coast and the Qinghai‐Tibet Plateau and Xinjiang. Over the study period, the Northeast China Plain gradually playing an increasing dominant role in grain output (Figure 6a). The meat products were mainly flowing from the central‐eastern regions to the country's major urban agglomerations, with an increasingly flowing trend nationwide (Figure 6b). The vegetables flows mirrored the grain's pattern, while some northwestern areas like Xinjiang emerging as notable output regions (Figure 6c). Water yield flows remained stable, originating in river headwaters (e.g., Qinghai, upper Heilongjiang) and moving toward the North China Plain and Xinjiang (Figure 6d). Carbon sequestration flows were dominated by northwestern China and the North China Plain as primary sink regions meeting demand from elsewhere (Figure 6e). Air purification was overall transferred to Xinjiang and the North China Plain, with the flow‐in area in the North China Plain shrinking (Figure 6f). Habitat quality flow closely tracked human settlement patterns along the Hu Huanyong Line, flowing from the northwest to the southeast (Figure 6g). Tourism recreation flows originated in metropolitan centers and key scenic spots and expanded in intensity to other regions nationwide, with a surge in flowing intensity nationwide (Figure 6h).

**FIGURE 7:**
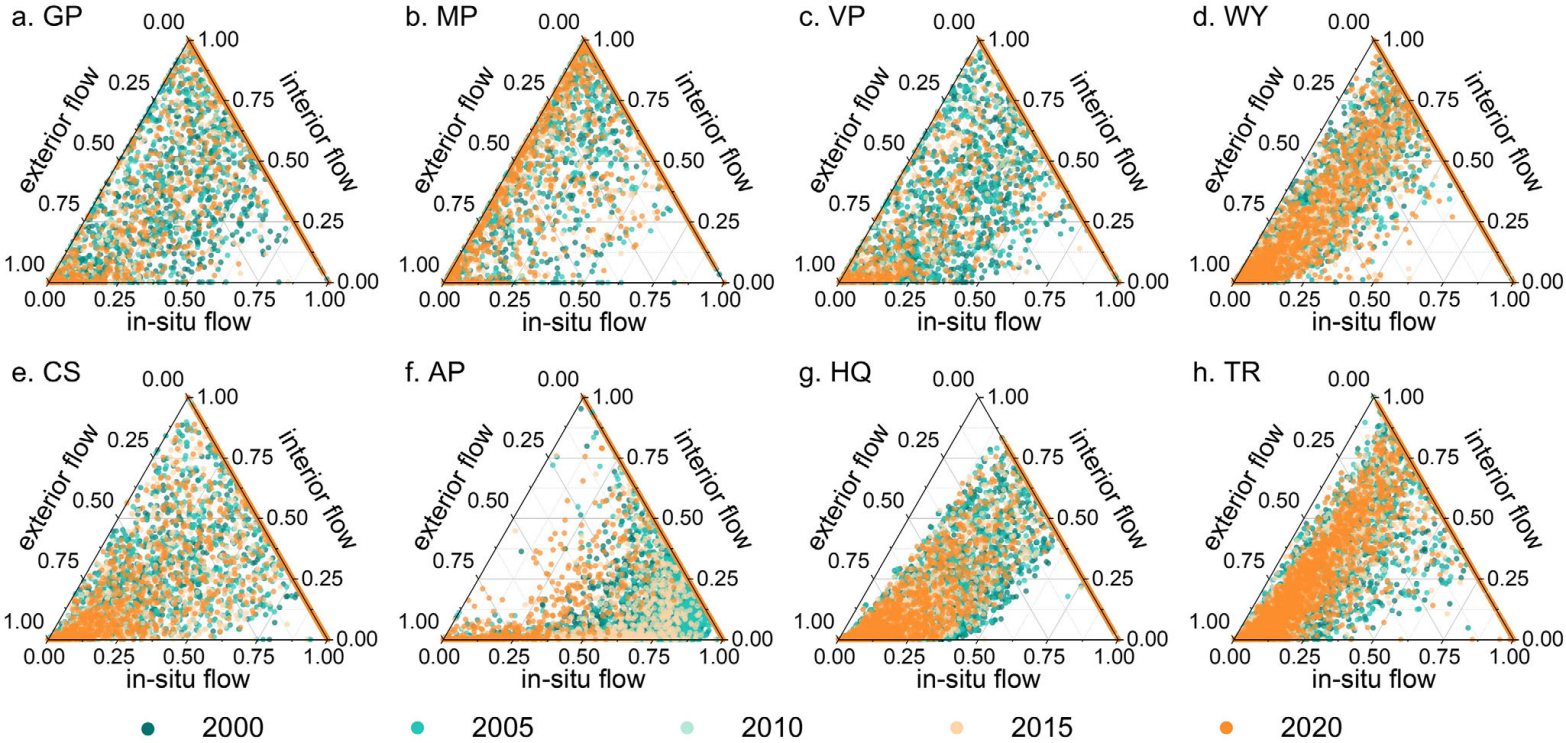
Ternary diagram of ESF structure in China. Panels a–h represent eight ecosystem services included in ESF assessment: (a) grain production (GP), (b) meat production (MP), (c) vegetable production (VP), (d) water yield (WY), (e) carbon sequestration (CS), (f) air purification (AP), (g) habitat quality (HQ), and (h) tourism recreation (TR). County‐scale ternary distributions of in situ flow (ISF), interior flow (IF), and exterior flow (EF) for eight ES categories from 2000 (dark green) to 2020 (orange). Each point represents one county's ESF composition (n = 2846 administrative units). Statistical testing was not performed, as the distribution represents the full census of counties in the study area.

County‐scale ternary plots reveal that point clouds migrated slightly toward the exterior‐flow (EF) apex over the two decades, signaling a growing reliance on cross‐county transfers (Figure 7). Specifically, the nationwide average proportions of ISF, IF, and EF for grain shifted from 48:45:7 to 40:44:16. Although most counties remained dominated by ISF and IF, the marked rise in EF indicates a decline in local grain self‐sufficiency and a gradual shift toward long‐distance transport to alleviate spatial supply–demand mismatches (Figure 7a) Meat's nationwide ISF∶IF∶EF shifted from 9∶65∶26 to 7∶55∶38. At the county scale, meat flows evolved from being IF‐dominated to a mix of IF and EF, reflecting a transition from primarily intra‐county circulation toward both intra‐county and inter‐county circulations (Figure 7b). Vegetable's nationwide ISF∶IF∶EF shifted from 49∶44∶7 to 41∶44∶15. County‐scale vegetable flows mirrored grain's pattern, with long‐distance transport gradually supplanting local self‐sufficiency (Figure 7c). Water yield's nationwide ISF∶IF∶EF shifted from 16∶30∶54 to 19∶31∶50. Counties displayed a slight migration toward ISF, indicating a gradual enhancement of local water provisioning capacity (Figure 7d). Carbon sequestration's nationwide ISF∶IF∶EF shifted from 44∶26∶30 to 26∶22∶52. County‐scale ternary distributions show an increasing number of counties dominated by EF, indicating growing reliance on remote carbon sinks to absorb local excess emissions (Figure 7e). Air purification's nationwide ISF∶IF∶EF remained relatively stable from 2000 to 2015 (37∶7∶56 to 40∶8∶52) but then shifted sharply to 6∶4∶90 by 2020. At the county level, improved air quality led to a drastic reduction in local purification demand, leaving only a handful of counties still reliant on EF (Figure 7f). Habitat quality's nationwide ISF∶IF∶EF shifted marginally from 30∶13∶57 to 29∶16∶55. County‐scale flows remained largely stable, with EF consistently predominant across most counties (Figure 7g). Tourism recreation's nationwide ISF∶IF∶EF shifted from 14∶19∶67 in 2000 to 19∶29∶51 in 2020. At the county scale, flows stayed EF‐dominated. However, the proportion of counties relying more on IF and ISF increased, reflecting a shift toward combining local short‐distance and regional long‐distance tourism modes (Figure 7h).

### 3.4 Ecosystem Management Zoning

**FIGURE 8:**
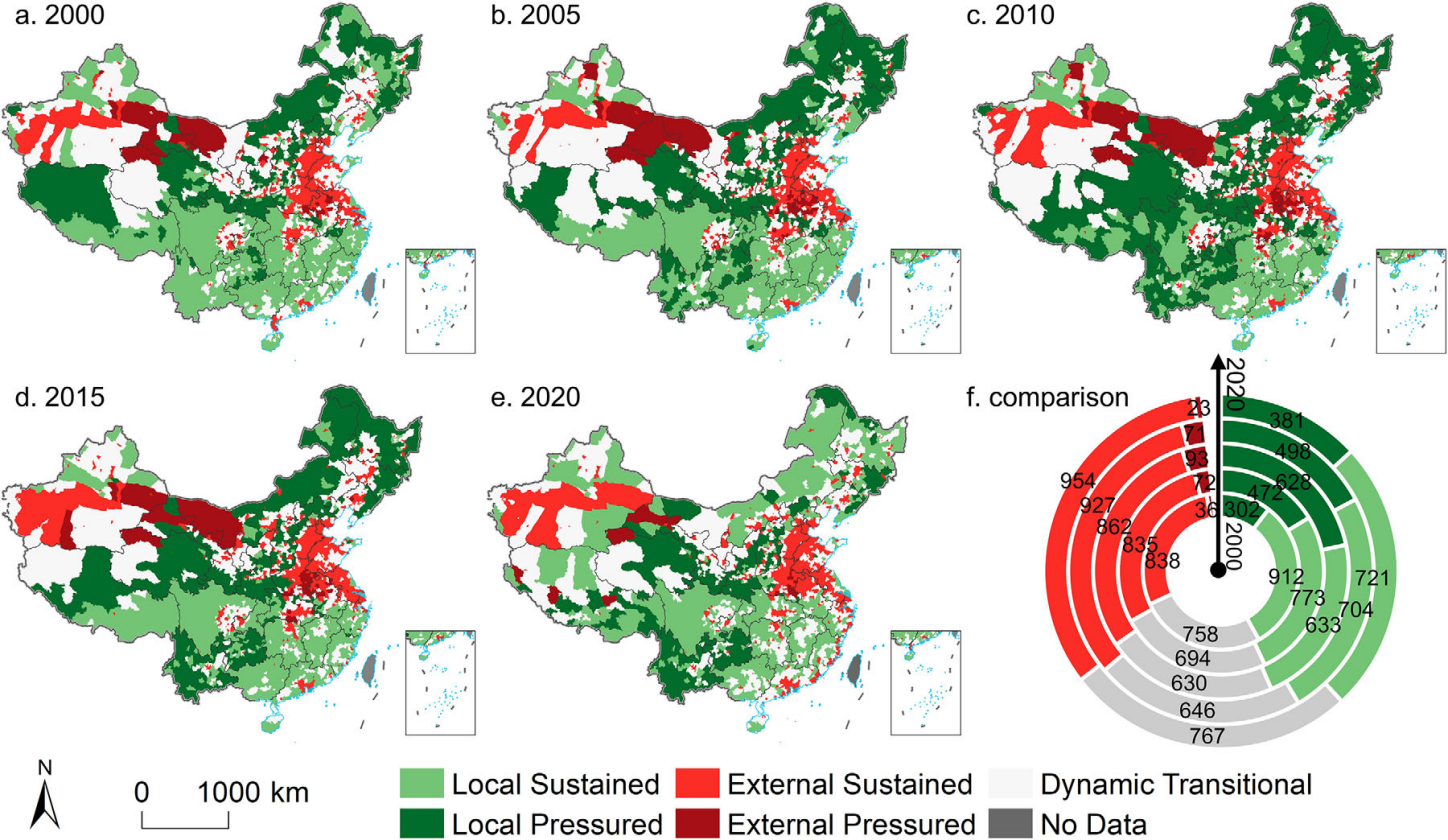
Dynamics of ecosystem management zoning in China. Results are aggregated and presented at the county level (n = 2846 administrative units). Data represent the absolute quantified state of the entire study area; thus, frequentist *p* values and significance testing are not applicable to this mapping approach.

Five ecosystem management zones were identified based on county‐scale demand fulfilment and flow structure: local sustained, local pressured, external sustained, external pressured, and dynamic transitional (Figure 8). Overall zoning remained stable but exhibited notable regional adjustments. For quantity trend, local sustained counties declined steadily (from 32.0% to 25.3%), with many shifting toward dynamic transitional (around 22.1% to 27.0%) and external sustained (from 29.4% to 33.6%). Local pressured counties first increased (from 10.6% in 2000 to 22.1% in 2010) and then decreased (from 22.1% in 2010 to 13.4% in 2020), indicating a transition from disorder to re‐coupling (Figure 8f). For the Baseline pattern (2000), local sustained counties clustered in humid southern China; local pressure in the Northeast, Qinghai–Tibet, and southwest mountains; external sustained in the North China Plain; external pressure in the arid northwest; dynamic transitional scattered nationwide (Figure 8a). For spatial evolution, in the Northeast and parts of North China, local pressured counties shifted to local sustained as agricultural pressure eased. On the Yunnan–Guizhou Plateau, local sustained counties became local pressured due to rising socio‐economic demand. Along the southeastern coast, local sustained counties evolved into external sustained, reflecting increased reliance on external ESs (Figure 8b–e).

## 4 Discussion

### 4.1 Reliability of the Assessment Results

The reliability of this study is better evaluated at the level of macro‐scale spatial patterns and interregional contrasts rather than pixel‐level absolute precision. Quantitative cross‐validation results for the PS of three provisioning services showed high consistency between provincial totals aggregated from county‐level estimates and official statistics (Figure S1). Spatial pattern comparison for most ES indicators also exhibited moderate to high agreement, although the PS of SR and the PD of WY, SR, and CS exhibited partial inconsistencies (Tables S6 and S7). Such inconsistencies are expected given the differences in modelling frameworks, proxy indicators, and conceptual definitions of ES supply and demand across studies. Nevertheless, the general spatial agreement indicates that the assessment captures the major geographical gradients of ES supply and demand.

The algorithmic verification further supports the internal credibility of the framework. The inter‐county allocation satisfied the key accounting constraints, including non‐negativity of transfer flows, the absence of simultaneous inflow and outflow for the same county, and national closure between AS and AD (Table S8). In addition, the ESF decomposition remained fully consistent, with total ESF equal to the sum of ISF, IF, and EF and EF matching the inter‐county transfer matrix (Table S9). These results indicate that the derived variables (AS, AD, and ESF) are internally coherent within the framework, providing a necessary basis for interpreting the subsequent flow analysis and zoning results.

Sensitivity tests indicate that the zoning results are relatively stable across moderate threshold variations. Adjusting the classification threshold within the tested range (±0.05–±0.15) resulted in only minor changes in agreement with the baseline scheme (average agreement ≈ 0.87), while the ±0.10 specification achieved a more balanced trade‐off between temporal stability and spatial discrimination (Tables S11–S13). In contrast, excluding ecosystem service indicators led to a more pronounced reduction in similarity to the baseline zoning, particularly when both HQ and TR were excluded (Tables S11–S13). This indicates that zoning outcomes are more sensitive to indicator composition than to moderate threshold variation, suggesting that removing ES indicators may substantially alter the representation of ecological and cultural functions in the zoning scheme.

### 4.2 Spatial Mismatches Between ES Supply and Demand

**FIGURE 9:**
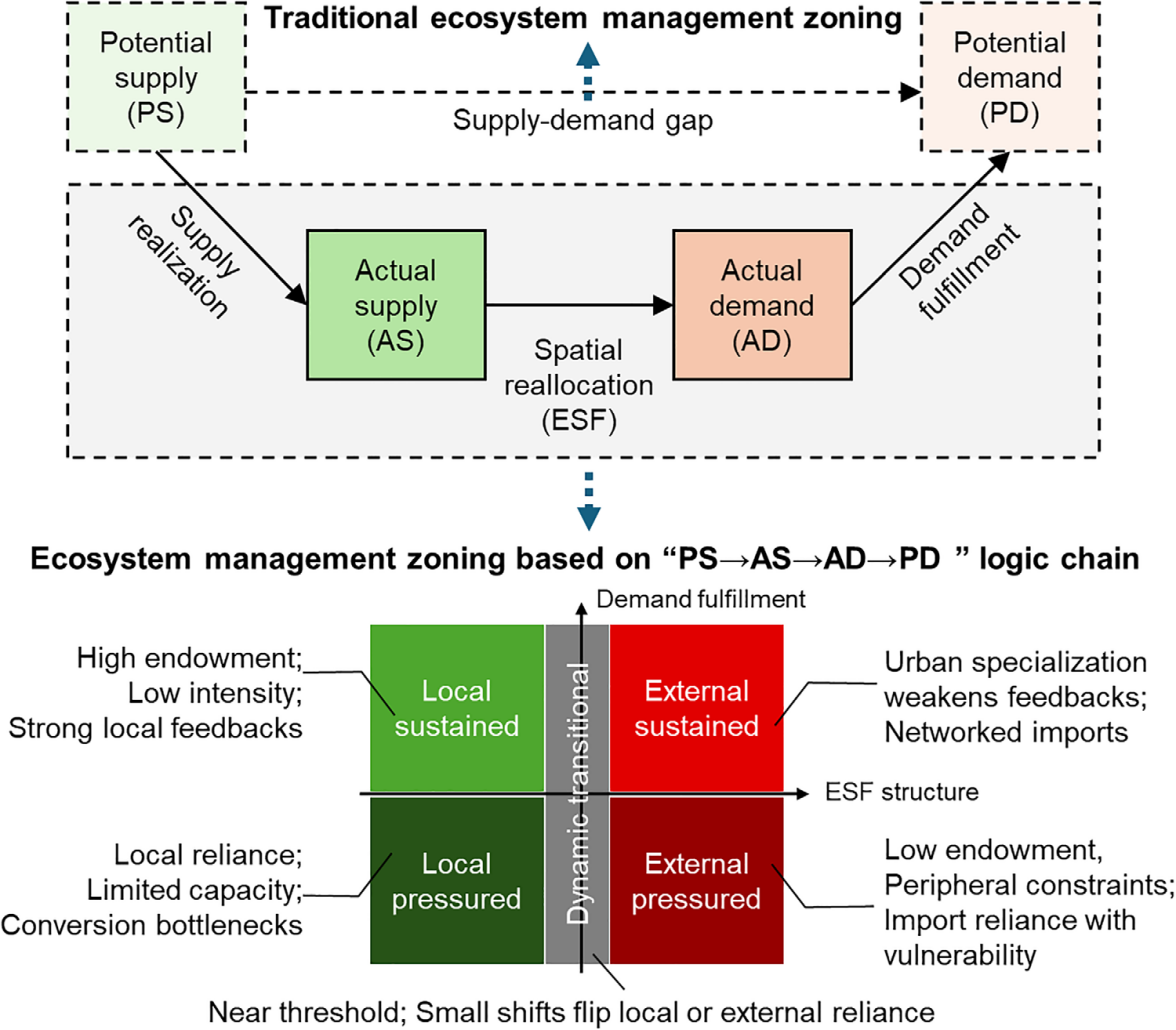
Decomposition and zoning logic under the supply–demand–flow framework. Note: Building on the theoretical framework in Figure 1, the supply–demand–flow framework was unpacked via the PS→AS→AD→PD chain. Arrows showed analytical reading only; they did not indicate the estimation order or imply causality.

According to the dynamics between potential supply (PS) and potential demand (PD), spatial mismatches in material products and cultural services were observed in metropolitan regions and dominant agricultural zones, whereas mismatches for regulating services occur mainly in the Northeast, the Tibet–Qinghai Plateau, and Xinjiang (Figures 2 and 3). This overall pattern was broadly consistent with existing studies [40, 41, 42]. Nevertheless, most previous studies barely selected the gap between PS and PD for ecological zoning [7, 42, 43]. Although previous zoning logic answered whether potential supply could meet standardized demand theoretically, it failed to tell whether the mismatches were driven by unrealized capacity or insufficient capacity. To address this “black box”, we decomposed the PS→PD relation into three measurable subprocesses: Supply realization (PS→AS), Spatial reallocation (AS→AD), and Demand fulfillment (AD→PD) (Figure 9). This process view turns a static “mismatch” into a set of actionable levers, in line with emerging work that separates generation, movement, and use of ESs [6].

In terms of supply realization (PS→AS), lower PS‐to‐AS ratios were observed in the Northeast (GP, VP), in the early‑period Southwest (MP), and across Southern China (SR, CS) (Figures 2 and 4). However, a lower realization ratio does not imply technical inefficiency. For material products, the Northeast and the early period Southwest remained a low realization ratio despite high output. The shortfall reflected limited local absorption driven by population density and consumption patterns, indicating a structural producer–consumer mismatch rather than process failure [44, 45]. For regulating services, high biophysical potential in the South sits alongside low standardized need, resulting in a low PS‐to‐AS ratio despite strong underlying capacity [46]. Conversely, high realization is insufficient for security if demand fulfillment remains below unity (e.g., AP). In such a case, the full mobilization of available capacity still leaves a residual demand gap [47]. Therefore, an integrated assessment of both the realization ratio and the subsequent capacity to meet demand is essential for accurately diagnosing mismatches in this process.

In terms of spatial reallocation (AS→AD), which captures the geographical realization of ESF by connecting supply areas with beneficiary locations. Unlike the potential supply and demand (PS‐PD) mismatch, it emphasizes the actual redistribution pathways of services. The AS–AD mismatch is largely structural. For example, HQ exhibits clear contrasts across the Hu Huanyong line due to physiographic constraints; TR reflects the heterogeneous distribution of tourism resources and preference patterns [29, 40]. Policies and engineering can modulate AS–AD gaps to some extent. For instance, the WY mismatch between AS and AD appears smaller than the PS–PD gap, plausibly reflecting the influence of large‐scale water transfer infrastructures such as the Three Gorges Project and the South‐to‐North Water Diversion Project [48, 49].

In terms of demand fulfillment (AD→PD), we applied AD/PD to characterize whether actual acquisition meets standardized demand. A ratio equal to or greater than 1 indicates demand is met or exceeded, while a ratio below 1 indicates either supply constraints or limitations arising from preferences and institutions. In our study, SR and AP exhibit relatively low fulfillment, consistent with constrained potential supply or limited realization [15, 47]. For TR, fulfillment is initially low in rural counties but rises over time, suggesting that latent capacity existed yet was underutilized in the early stages and subsequently activated as county‐level tourism developed [29, 50]. This interpretation is congruent with ES accounting frameworks that distinguish supply from use, and with evidence that cultural and tourism services are sensitive to shifting urban–rural preferences and local development strategies [51, 52].

### 4.3 Influencing Mechanisms of ESF

In this study, we categorized ESFs into in situ flow (ISF), interior flow (IF), and exterior flow (EF), following prevailing definitions in the ESF and metacoupling frameworks [15, 16, 53]. This classification provides a dual perspective for management, it not only identifies which counties act as net suppliers or net recipients of ES surpluses, but also identifies the degree to which counties rely on local ecological feedbacks (high ISF and IF) or distant linkages (high EF). At the national scale, the overall spatial pattern of ESF has remained relatively stable over the past two decades (Figure 6), reflecting the inertia of geographic endowments, socio‐economic configurations, and preference landscapes. By contrast, the internal composition of flows—namely the relative shares of ISF, IF, and EF—has shifted noticeably (Figure 7), suggesting differentiated mechanisms of change.

The structural shifts of ESF can be interpreted through three types of drivers: natural, social, and the co‐influence of natural and social. HQ is primarily shaped by physiographic gradients, most prominently across the Hu Huanyong Line, and thus its flow structure remains highly stable with limited responsiveness to social change [54, 55]. By contrast, provisioning services such as GP, MP, and VP reveal modest declines in ISF and IF, and corresponding increases in EF, reflecting improvements in long‐distance transportation and evolving dietary preferences, particularly for animal‐based products [56, 57]. Cultural services show the opposite trend with reduced external dependence and increased local inflows, which reflects the expansion of county‐scale tourism and a rebalancing of preferences toward nearby recreations [50]. Regulating services such as WY, CS, and AP exhibit mixed dynamics, jointly influenced by natural and social processes [42]. For instance, the growing share of local WY flows reflects interactions between natural retention capacity and large‐scale storage or diversion projects. By enhancing diverse habitat mosaics, the local habitat ecological feedbacks and overall system resilience can be strengthened [58]. Meanwhile, increasing external dependence of CS and AP is associated with heterogeneous land‐use change, vegetation dynamics, and human pressures that redistribute regulating benefits across space.

From a management perspective, recognizing whether a county relies primarily on local or exterior flows is necessary but insufficient. A county characterized by high exterior inflows coupled with sufficient demand fulfillment may represent a stable comparative advantage, whereas high external dependence combined with unmet demand exposes vulnerabilities to external shocks [6, 59]. Only by further cross‐classifying the flow ESF structure with the demand fulfillment ratio can we distinguish between sustained flows and pressured flows. This diagnostic framework provides an operational basis for zoning and informs the policy levers discussed in Section 4.3 (Figures 8 and 9).

### 4.4 Strategies for Ecosystem Management in China

Building on the overlay of the two policy‑proximal endpoints of the PS→AS→AD→PD chain, namely spatial reallocation and demand fulfillment, our management zoning exhibits a spatial gradient broadly consistent with the Green Loop‐Red Loop (GL‐RL) heuristic [60] (Figures 8 and 9). While ESF is often conceptualized through “servicesheds”—the areas providing services to specific beneficiaries—such ecological boundaries do not always coincide with administrative governance structures [33, 34]. In this study, the county was adopted as the core accounting unit to ensure that the supply–demand–flow diagnostics remain compatible with administrative decision‐making. Under this setting, the zoning framework helps reveal cross‐boundary ecological dependencies while remaining compatible with county‐level governance.

Consistent with the GL–RL heuristic proposed by Cumming, et al. [60], external sustained clusters are concentrated in highly urbanized regions where local ecological feedbacks are attenuated and distant couplings are strong, as illustrated by Jing‑Jin‑Ji and the southeastern coast. Recent work in China also highlighted that urban expansion can induce both local and remote ES losses, thereby reinforcing cities’ external dependence [61]. Local sustained areas are typically located in regions with relatively abundant ecological supply and lower socio‐economic intensity, which is characteristic of much of southern China. Transitions from local pressure to local sustained have also taken place through ecological restoration and eco‐economic investment, with notable examples in Northeast China. External sustained or external pressured counties are identified in the northwest despite low urbanization. Unlike findings from South Africa, where red‐loop conditions are mainly associated with urban fringes, the Chinese pattern is better explained by limited ecological supply rather than urban demand alone [62]. Generally, these findings are consistent with GL‐RL expectations in urban cores, where local feedback weaken and distant couplings strengthen [62, 63]. Beyond this, the results extend the heuristic by showing that in arid and sparsely populated regions, supply‐side constraints can also lead to red‐like states.

Regarding development trajectories, international evidence suggests that shifts toward stronger distant couplings often accompany rapid economic growth, and the external sustained zones in China do coincide with higher development levels [63]. However, high‑quality socio‑ecological outcomes are not limited to such pathways. With appropriate governance, local sustained regions can maintain strong local feedback while improving welfare. Emerging pilots on ecological‐product valuation and compensation, such as ecological bank initiatives in Fujian and ecosystem product practices in Zhejiang, illustrate this potential [64, 65, 66]. Accordingly, the GL‐RL framework is treated here as a heuristic point of reference rather than a prescriptive path, with the desirable endpoint being a resilient configuration adapted to local ecological endowments and institutional capacities.

For China's ecosystem management, the key strategies for different zones can be summarized as follows. Local sustained areas should focus on maintaining ecological surpluses and strengthening mechanisms for ecological product valuation and compensation. Local pressured areas should focus on improving conversion from potential to actual supply or reducing standardized demand through industrial restructuring and efficiency measures. Enhancing agricultural management practices, including maintaining soil nutrient balance and organic carbon stocks, is also essential for sustaining regulating services and long‐term ecosystem performance [67]. External sustained areas should formalize and stabilize cross‑regional compensation and governance arrangements to secure the reliability of external inputs. External pressured areas should reduce vulnerability by combining targeted ecological restoration with local capacity building to lessen exposure to distant shocks. Dynamic transitional areas should adopt threshold‑based monitoring and early warning protocols to prevent slippage toward vulnerable external dependence or unmet demand states.

### 4.5 Limitations and Future Prospects

This study applies a scale‐based “supply–demand–flow” perspective to classify ESF and support regional ecosystem management. Nevertheless, several limitations should be acknowledged. First, our study adopts the county as the primary management unit. While this scale aligns with existing ecological governance frameworks, ESFs are shaped by ecological processes that may extend beyond administrative boundaries. Second, the conceptual framework simplifies certain real‐world dynamics. In particular, cross‐border ESFs beyond the national boundary and potential losses during the transfer from actual supply to actual demand are not explicitly modeled. Third, uncertainty remains in both the quantification of ecosystem services and the resulting zoning outcomes. Several ecosystem services are quantified using proxy indicators due to data limitations, particularly for cultural services. In addition, some modeling choices, including parameter settings in biophysical and socio‐economic models and the selection of zoning thresholds and service weights, may influence the zoning results. Nevertheless, sensitivity analyses suggest that the main spatial patterns of ESFs and the resulting zoning scheme remain relatively stable under moderate parameter variation.

Looking ahead, future research can build on the existing analytical framework in several directions. On the research side, integrating network or metacoupling metrics would better represent multi‐scale origin–beneficiary paths and explicitly consider reliability, risk, and equity in who bears unmet demand [53, 68]. On the practice side, the five‐zone typology can be embedded in territorial‐spatial governance and aligned with dual‐carbon tasks, supported by pilot dashboards that regularly track realization, reallocation, and fulfillment indicators [69]. Together, these extensions would strengthen both the explanatory capacity and the practical relevance of the framework, while maintaining its policy‐oriented focus.

## 5 Conclusion

This study developed and applied a “supply–demand–flow” framework to map the generation, redistribution, and fulfillment of ES across China from 2000 to 2020 to support differentiated ecosystem management. By decomposing the pathway from potential supply to potential demand into the subprocesses of supply realization, spatial reallocation, and demand fulfillment, our analysis moves beyond traditional static assessments and provides a process‐oriented perspective for diagnosing mismatches. The results revealed strong spatial heterogeneity and shifts in internal structure: most provisioning and regulating services became increasingly dependent on external flows, while water yield and tourism remained locally sustained. Based on flow balance and demand fulfillment, five county‐level management zones were identified, reflecting the diverse conditions across China. Overall, our framework improves conceptual clarity, demonstrates nationwide operationalization, and generates policy‐relevant zoning that can inform ecosystem management. It offers a practical tool for aligning ecological functions with management priorities and provides transferable insights for global efforts to reconcile development and conservation in pursuit of sustainability.

## 6 Methods

### 6.1 ES Supply and Demand Measurement

#### 6.1.1 Indicator Selection

Considering the completeness and the representativeness of the accounting system, nine indicators categorized in three dimensions of ecological products were selected in this study (Table 1). As supporting services cannot directly benefit economic systems, only material products, regulating services, and cultural services were included [70, 71]. Specifically, grain, meat, and vegetables consumed by residents in their daily lives were selected as indicators for measuring material products. Water yield, soil retention, carbon sequestration services, air purification, and habitat quality were selected as indicators for evaluating the function of the ecosystem's regulating services dominated by the factors of water, soil, atmosphere, and biology. Recreational tourism was selected as an indicator for measuring cultural services [29, 71, 72].

**TABLE 1:** Measurement indicator system.

Dimension	Indicator	Acronym
Provisioning services	Grain production	GP
	Meat production	MP
	Vegetable production	VP
Regulating services	Water yield	WY
	Soil retention	SR
	Carbon sequestration	CS
	Air purification	AP
	Habitat quality	HQ
Cultural services	Tourism recreation	TR

#### 6.1.2 Measurement Methods

This study proposed an integrated three‐step framework to quantify ES supply and demand at the raster (1 km × 1 km) scale. (1) Potential supply quantification. From a biophysical perspective, the ARIES, RUSLE, i‐Tree, InVEST models, kernel‐density, and empirical functions with GIS spatial analysis are combined to quantify the theoretical maximum supply of each service under current land‐use and climatic conditions. (2) Potential demand quantification. From a socio‐economic perspective, we used data on population density, urban and rural per‐capita consumption rates, GDP, and sectoral resource‐use statistics (e.g., agricultural and industrial water withdrawals, carbon emission quotas, PM_2_._5_ concentrations) to quantify per‐capita or per‐area needs and spatialize these needs via GIS to quantify the potential demand for each ES. (3) Actual supply‐demand quantification. First, we use socio‐economic data (population density, urban and rural per‐capita consumption, sectoral water withdrawals, carbon emission quotas, etc.) with GIS spatial analysis to quantify raster‐level actual demand (AD_i_); then, following the “local consumption + external compensation” logic, grid‐level ESF between actual supply (AS_i_) and demand (AD_i_) locations are allocated through the Iterative Proportional Fitting (IPF) procedure. The detailed allocation process and constraints are described in Section 6.2.

The detailed equations and indicator definitions for each service category: material products (GP, MP, VP), water yield (WY), soil retention (SR), carbon sequestration (CS), air purification (AP), habitat quality (HQ), and tourism recreation (TR) are listed in Table S1.

### 6.2 Measurement of ESF

ESF fundamentally represents the spatial reallocation of actual ES supply (AS) to satisfy actual societal demand (AD) [16, 73]. In the accounting of realized ecosystem service transfers, this study adopts a closed system‐scale accounting framework, such that the total actual supply equals the total actual demand (ΣAS = ΣAD). Potential losses during the transfer process are not explicitly modeled in this national‐scale assessment.

(1) $$\sum ESF=\sum AS=\sum AD=\min\left(\sum PS,\;\sum PD\right)$$

where *PS* and *PD* are potential ES supply and demand. The minimum of potential supply and demand defines the upper bound of ESs that can be realized within the coupled socio‐ecological system.

Building on Equation (1), the total ESF can be further decomposed. As defined in Section 2:

(2) ESF=ISF+IF+EF

where *ISF*, *IF*, *EF* are in situ flow, interior flow, and exterior flow, respectively.

ISF represents the self‐sufficient portion of ESs. Following the assumption that local demand is satisfied prior to export [74], the ISF equals the sum across all grid cells of the minimum of supply and demand in each cell:

(3) $$ISF=\sum \min\left(PS,PD\right)$$

IF focuses on ecosystem‐service transfers that occur outside individual grid cells but within the same county. The total volume of services available within a region is min(∑PS,∑PD); subtracting the self‐sufficient ISF:

(4) $$IF=\min\left(\sum PS,\sum PD\right)-ISF$$

EF focuses on ecosystem‐service flows that cross regional (county) boundaries. According to Equation (2), EF can be calculated as:

(5) EF=ESF−ISF−IF

Compared with traditional approaches to measuring ESF, our framework accommodates both the quantitative magnitude and spatial relationships of services while requiring relatively modest data inputs. Moreover, Equations (2)–(4) can be applied at multiple spatial levels, enabling multi‐scale ecosystem management. Specifically, for soil retention, although it is often treated as an in situ regulating service in ecosystem accounting frameworks [75], sediment transport processes may generate downstream benefits along river systems, as demonstrated by recent studies on soil conservation service flows [76]. In this study, soil retention is therefore treated as a predominantly in situ service, and cross‐regional flows are not explicitly modeled at the national scale.

### 6.3 Zoning Method for Ecosystem Management Based on ES

To support spatially explicit management, this study classified each county into one of five ecological‐management zones according to two key indicators: (1) Flow balance index (P_ISF_‐ P_EF_), defined as the difference between the share of in situ flow and the share of exterior flow, where the denominator for both shares is the total ESF volume (ISF+IF+EF) within the county boundary; (2) Demand fulfillment ratio (AD/PD), calculated as the county‐level actual demand divided by potential demand. The two indicators were averaged across the eight flow‐enabled service categories [6], following previous studies that synthesize multiple ecosystem services using arithmetic aggregation to represent overall service performance. Drawing on the classification logic of Liu, et al. [77], this study partitioned the counties into five management categories according to threshold values (Table 2). The threshold value (±0.10) was selected to balance spatial differentiation and classification stability, and its robustness was further evaluated through sensitivity tests (Section 6.4.3).

**TABLE 2:** Zoning Categories for County‐Level Ecosystem Management.

Categories	Description
Local Sustained	P_ISF_‐ P_EF_≥0.1 and AD/PD≥1. Counties whose local supply comfortably meets demand.
External Sustained	P_ISF_‐ P_EF_≤‐0.1 and AD/PD≥1. Counties that rely on imports but maintain overall supply–demand balance.
Local Pressured	P_ISF_‐ P_EF_≥0.1 and AD/PD≤1. Counties with insufficient internal supply relative to demand.
External Pressured	P_ISF_‐ P_EF_≤‐0.1 and AD/PD≤1. Counties both import‐dependent and demand‐exceeding, indicating unsustainability.
Dynamic Transitional	|P_ISF_‐ P_EF_|≤0.1. Counties in a transition state, neither strongly self‐sufficient nor wholly import‐dependent, regardless of demand ratio.

### 6.4 Verification and Robustness Checks

#### 6.4.1 Consistency and Plausibility Assessment

In this study, 9 ES indicators were derived using different data sources and modelling approaches; a single uniform validation procedure could not be applied to all indicators. Moreover, existing studies often adopt different modelling approaches or proxy indicators and do not consistently distinguish between potential and actual supply or demand, which limits the comparability of numerical estimates across studies [78, 79]. Quantitative cross‐validation was therefore conducted only for three provisioning services (GP, VP, and MP) by aggregating the downscaled PS to the provincial level and comparing it with official statistical records (Figure S1). More generally, all ES indicators, including both PS and PD, were assessed for plausibility by comparing their spatial patterns with those of previous studies. Specifically, we examined whether the simulated spatial distributions reproduced well‐established geographical gradients and service mechanisms reported in the literature (Tables S6 and S7).

#### 6.4.2 Algorithmic Verification

Since AS, AD, and ESF were derived through an IPF‐based inter‐county allocation framework, their validation focused on internal consistency rather than external calibration. Specifically, we examined whether the balanced transfer matrix linking AS and AD satisfied the required accounting constraints, including non‐negativity of inter‐county transfers, the absence of simultaneous non‐zero inflow and outflow for the same county, and national closure between aggregated AS and AD (Tables S8). For the ESF consistency verification, we checked whether the total ESF should equal the sum of ISF, IF, and EF and whether EF remained fully consistent with the inter‐county transfer matrix derived from the IPF procedure (Tables S9).

#### 6.4.3 Robustness and Sensitivity Analysis

The sensitivity analysis of the zoning scheme was evaluated on three dimensions. Cross‐sectional differentiation was assessed for each scenario and year by reporting the number of counties assigned to each zoning type and the proportion of the largest class (largest class share), indicating the degree of spatial differentiation in the zoning outcome [80]. Temporal stability was evaluated at the county level across the five study years using two indicators: the dominant‐class share, defined as the proportion of years in which a county remained in its most frequent zoning type [81], and the transition rate, defined as the proportion of interannual transitions in which the zoning type changed [82]. Similarity to the baseline specification (S0) was measured as the proportion of counties whose zoning type matched the baseline classification for each year, indicating the extent to which alternative specifications altered the spatial structure of the zoning outcome [83]. Because the analysis compares complete zoning results for all counties rather than estimating parameters from a sample, statistical significance testing was not applicable; robustness was therefore assessed through structural stability and agreement metrics.

### 6.5 Data Sources

The data used in this study are grouped into three categories: raster data, vector data, and socio‐economic data. All raster data was unified to 1 km × 1 km resolution, with a time series spanning 2000–2020, while the vector data mainly include tourism POIs and county administrative boundaries. The tourism POIs represent the spatial distribution of tourism resources and are treated as time‐invariant, whereas temporal dynamics of tourism recreation services were reconstructed using annual tourism statistics. The administrative boundary data were used to ensure a consistent spatial framework for aggregating ecosystem service indicators and conducting zoning analysis (Table 3).

**TABLE 3:** Data Sources and Description.

Attribute	Data	Sources and Description
Raster dataset	LUCC	China Multi‐Period Land Use Land Cover Remote Sensing Monitoring Dataset [84] (http://www.resdc.cn/)
	Population raster data	LandScan Global Population Database (http://www.resdc.cn/)
	GDP raster	Kilometer raster dataset of China's GDP spatial distribution (http://www.resdc.cn/)
	Carbon emission raster	The Open‐Data Inventory for Anthropogenic Carbon dioxide (ODIAC) (https://cger.nies.go.jp/en/)
	Nighttime light	An extended time‐series (2000‐2023) of global NPP‐VIIRS‐like nighttime light data (https://dataverse.harvard.edu/)
	DEM	National Geospatial Data (http://www.gscloud.cn/sources)
	NDVI	Terra MODIS NDVI data [85]
	NPP	MODIS_MOD17A3 data [86]
	PM_2.5_	Third Pole Environment Data Center [87]
	Precipitation	National Earth System Science Data Center (http://www.geodata.cn/)
	Evapotranspiration	National Tibetan Plateau Data Center [88] (https://data.tpdc.ac.cn/)
	Soil Database	Harmonized World Soil Database v1.2 (HWSD), (http://webarchive.iiasa.ac.at/Research/LUC/External‐World‐soil‐database/HTML/)
Vector dataset	POI	By geographic inverse coding A‐level scenic spots obtained from China's ministry of culture and tourism (https://zwfw.mct.gov.cn/wycx/5ajlyjq/)
	Administrative areas data	County administrative areas data [89] (http://www.resdc.cn/)
Socio‐economic dataset	Statistical yearbook data	Production and consumption of grains, vegetables, meat, tourism revenue, and water resources bulletin data (2000‐2020) (https://www.stats.gov.cn/sj/ndsj/) (http://www.mwr.gov.cn/sj/tjgb/szygb/)
	Model parameters	Model parameters for ecological products (see supplementary information)

### 6.6 Statistical Analysis

All spatial data processing and visualizations were conducted using ArcMap 10.7, Python 3.11, and Origin 2024. Pre‐processing focused on spatial alignment and consistency, whereby all datasets were projected to a unified coordinate system and resampled to a 1 km spatial resolution. To ensure spatial continuity, any NoData values within the study area boundary were treated as zero; notably, no data transformation or normalization was applied to preserve the original biophysical units of the ESs. This study employs a full‐census approach, with the sample size (n) consisting of approximately 9.5 × 10^6^ pixels for grid‐based mapping (Figures 2, 3, 4, 5) and 2844 administrative county units for the aggregated analysis of ESF and management zoning (Figures 6, 7, 8). As the datasets represent complete spatial coverage (wall‐to‐wall mapping) of the defined region rather than random samples, inferential statistical tests (e.g., P values) were not required to describe the spatial distributions and structural compositions.

## Funding

This paper was supported by the National Key R&D Program of China (Nos. 2024YFD1600700 and 2024YFD1600701), National Natural Science Foundation of China (grant number 42271275).

## Conflicts of Interest

The authors declare no conflicts of interest.

(1) The LUCC data used for the basic calculation of ES supply and demand are available from the China Multi‐Period Land Use Land Cover Remote Sensing Monitoring Dataset [84] (https://www.resdc.cn/DOI/doi.aspx?DOIid = 54). Please note that the website is available only in Chinese; English readers may need to use translation tools to navigate. The data can be accessed and downloaded after registering for a user account.

(2) The production and consumption of grains, vegetables, and meat for material product supply and demand are available from Chinese statistical yearbook data (https://www.stats.gov.cn/english/Statisticaldata/yearbook/), including the editions for 2021, 2016, 2011, and 2006. The material product production data can be accessed in the table “Agriculture – Output of Major Farm Products”. The material product consumption data can be accessed in the tables “People's Livelihood – Per Capita Consumption of Major Food in Rural Households by Region” and “People's Livelihood – Per Capita Annual Consumption Expenditure of Urban Households by Region”. Please note that the 2001 yearbook (for the year 2000) is only available in Chinese (https://www.stats.gov.cn/sj/ndsj/2001c/mulu.htm); English readers may need to use translation tools to navigate.

(3) The precipitation and evapotranspiration data used for water yield supply are available from the National Tibetan Plateau Data Center [88] (https://www.tpdc.ac.cn/en/data/faae7605‐a0f2‐4d18‐b28f‐5cee413766a2/; https://data.tpdc.ac.cn/en/data/8b11da09‐1a40‐4014‐bd3d‐2b86e6dccad4/). These datasets can be directly downloaded from the pages. The by‐industrial water consumption data used for water yield demand are available from the National Water Resources Bulletin (http://www.mwr.gov.cn/sj/tjgb/szygb/), specifically for the years 2000, 2005, 2010, 2015, and 2020. The original data are published in PDF reports, and the values used in this study were manually extracted from the corresponding statistical tables. Please note that the bulletins are available only in Chinese; English readers may need to use translation tools to navigate.

(4) The DEM data used for the soil retention service are available from the National Geospatial Data Cloud (https://www.gscloud.cn/sources/details/306?pid = 302). The website is available only in Chinese, and the data can be accessed after registering for a user account. The soil attribute data are obtained from the Harmonized World Soil Database v1.2 (HWSD) (http://webarchive.iiasa.ac.at/Research/LUC/External‐World‐soil‐database/HTML/), where the database files can be downloaded directly.

(5) The NPP data used for carbon sequestration supply are from MODIS_MOD17A3 data [86] (https://www.earthdata.nasa.gov/data/catalog/lpcloud‐mod17a3hgf‐061). The data can be accessed through the “Data Access—OPENDAP DATA” option. The Carbon emission data used for carbon sequestration demand are available from Open‐Data Inventory for Anthropogenic Carbon dioxide (ODIAC) (https://www.nies.go.jp/doi/10.17595/20170411.001‐e.html), where the database files can be downloaded directly.

(6) The PM_2.5_ data used for air purification are available from the Third Pole Environment Data Center [87] (https://data.tpdc.ac.cn/en/data/6168e75d‐93ab‐4e4a‐b7ff‐33152e49d0bf/), where the database files can be downloaded directly.

(7) The 5A‐level scenic spots POIs and tourism revenue for tourism recreation are available from the Ministry of Culture and Tourism of China (https://zwfw.mct.gov.cn/wycx/5ajlyjq/; only available in Chinese) and Chinese statistical yearbook data (https://www.stats.gov.cn/sj/ndsj/2021/indexeh.htm), which can be accessed in the tables “Hotels, Catering Services and Tourism– Domestic Tourism”. Notably, city‐level tourism revenue data were manually collected from the publicly released National Economic and Social Development Statistical Bulletins of over 300 prefecture‐level cities and from the provincial statistical yearbooks of China. Most of them are only available in Chinese; English readers may need to use translation tools to access the information.

(8) Other socio‐economic data used for various ES demand and flow are obtained from the Resource and Environmental Science Data Platform (RESDC), including population raster data (https://www.resdc.cn/DOI/DOI.aspx?DOIID = 32), GDP raster data (https://www.resdc.cn/DOI/DOI.aspx?DOIID = 33), and administrative boundary data (https://www.resdc.cn/DOI/DOI.aspx?DOIID = 120). The RESDC website is available only in Chinese, and the data can be accessed after registering for a user account; English readers may need to use translation tools to navigate. The nighttime light data are obtained from the global NPP‐VIIRS‐like Nighttime Light dataset (https://doi.org/10.6084/m9.figshare.22262545.v8), where the database files can be downloaded directly.

## Supporting information




**Supporting File**: advs75284‐sup‐0001‐SuppMat.docx.

## Data Availability

The data used in this study are publicly available and were sourced from the following platforms:
