# Supplementary material for: Mapping the “Supply–Demand–Flow” of Ecosystem Services for Ecosystem Management in China
Source: Adv Sci (Weinh). 2026 Apr 15;13(39):e22070. doi: 10.1002/advs.202522070 (PMC13335012; doi:10.1002/advs.202522070)
Supplement: Supplementary file 1 — Supporting File: advs75284‐sup‐0001‐SuppMat.docx. [file ADVS-13-e22070-s001.docx]

Supporting Information

**Mapping the “Supply–Demand–Flow” of Ecosystem Services for Ecosystem Management in China**

*Yikun Zhang, Yongsheng Wang^*^, and Guirui Yu*

**Contents of this file**

Section Ⅰ ES quantification methods

Table S1. Methods for quantifying supply and demand of each ES category

Section Ⅱ Biophysical and socio-economic parameters for ES assessment

Table S2. Carbon sequestration demand measurement Indicators

Table S3. Average PM_2.5_ purification capacity for different land use types (t/km²)

Table S4. Parameters for habitat quality assessment

Table S5. Weights assigned to tourism-spot revenue levels

Section Ⅲ Consistency and plausibility assessment

Figure S1. Cross-validation of provisioning service supply

Table S6. Spatial validation of PS patterns through comparison with previous studies

Table S7. Spatial validation of PD patterns through comparison with previous studies

Section Ⅳ Algorithmic verification

Table S8. Algorithmic validation summary for the AS and AD allocation

Table S9. Algorithmic validation summary for ESF decomposition

Section Ⅴ Robustness and sensitivity analysis

Table S10. Scenario setting for sensitive analysis

Table S11. County number of zoning types and largest class share across scenarios

Table S12. Temporal stability metrics of zoning outcomes across scenarios

Table S13. Structural similarity between alternative scenarios and the baseline zoning

**Section Ⅰ ES quantification methods**

**Table S1.** Methods for quantifying supply and demand of each ES category

|  | Potential supply (PS) | Potential demand (PD) | Actual demand (AD) | Actual supply (AS) |
| --- | --- | --- | --- | --- |
| GP | Spatialized according the ratio of raster and total farmland NDVI value (grain, vegetable) or grassland NDVI value (meat) (Kuri et al., 2014; X. Zhang et al., 2022)  $\begin{aligned} {PS}_{k,i}={PS}_{k,sum}\times\frac{{NDVI}_{i}}{{NDVI}_{sum}} \end{aligned}$  Where ${PS}_{k,i}$ and ${PS}_{k,sum}$are the potential supply of provisioning service *k* (GP, VP, or MP) at pixel *i* and at the county scale, respectively;${NDVI}_{i}$ and ${NDVI}_{sum}$ are the farmland (or grassland) NDVI values of pixel *i* and of the entire county. | Potential demand for each provisioning service *k* at pixel *i* was quantified as the product of population density (${POP}_{i}$) with the nationally recommended per-capita dietary requirement ($R_{k}$):  ${PD}_{k,i}={POP}_{i}\times R_{k}$  Where the per capita grain demand was set at 118 kg/year, per capita meat demand at 22 kg/year, and per capita vegetable demand at 110 kg/year intake (Chinese Nutrition Society, 2022). | Based on per capita actual consumption of food, vegetables and meat in urban and rural areas in each province, combined with population data.  $\begin{aligned} {AD}_{k,i}={pop}_{i}\times\left[ {UC}_{k}\times UR+{RC}_{k}\times\left( 1-UR \right) \right] \end{aligned}$  Where ${AD}_{k,i}$ is the actual demand of provisioning services at the raster scale, ${pop}_{i}$ is the resident population density,${UC}_{k}$ and ${RC}_{k}$ are the per capita consumption of grain, vegetable, and meat in the urban and rural areas of the province where the raster is located, and $UR$ is the urbanization rate of the county where the raster is located. The county-level urbanization rate is calculated by (Y. Zhang & Wang, 2025). | According to the Iterative Proportional Fitting (IPF) algorithm of Input-Output analysis (Q. Chen et al., 2023; De Petrillo et al., 2025). At the county scale, the total actual supply of service 𝑘 in county *j* can be divided into an internal component that can be satisfied locally (${AS}_{k,j}^{I}$) and an external component that must be imported from other counties (${AS}_{k,j}^{E}$):  ${AS}_{k,j}={AS}_{k,j}^{I}+{AS}_{k,j}^{E}$  ${AS}_{k,j}^{I}=\min\left( \sum_{i=1}^{m_{j}} {PS}_{k,i},\sum_{i=1}^{m_{j}} {AD}_{k,i} \right)$  ${AS}_{k,j}^{E}=\max\left( 0,\sum_{i=1}^{m_{j}} {AD}_{k,i}-\sum_{i=1}^{m_{j}} {PS}_{k,i} \right)$  Where $m_{j}$ is the number of pixels in county 𝑗; ${PS}_{k,i}$ and ${AD}_{k,i}$ are potential supply and actual demand of the pixel-level.  At the raster scale, the actual supply of service 𝑘 at raster i can be represented as:  ${AS}_{k,i}={AS}_{k,i}^{I}+{AS}_{k,i}^{E}$  ${AS}_{k,i}^{I}=\min\left( {PS}_{k,i},{AD}_{k,i} \right)$  ${AS}_{k,i}^{E}=\rho_{j}\times\left( {PS}_{k,i}-{AS}_{k,i} \right)$  $\rho_{j}=\frac{{RS}_{k,j}-\sum_{i=1}^{m_{j}} \min\left( {PS}_{k,i},{AD}_{k,i} \right)}{{AS}_{k,j}-\sum_{i=1}^{m_{j}} \min\left( {PS}_{k,i},{AD}_{k,i} \right)}$  Where ${AS}_{k,i}$, ${AS}_{k,i}^{I}$ , and ${AS}_{k,i}^{E}$ are total actual supply of raster *i*, actual supply within raster *i*, actual supply from raster *i* to other rasters; $\rho_{j}$ is the realization rate of the county *j* from potential supply to actual supply.  Specifically, the soil retention can’t flow spatially, its actual supply (${AS}_{SR,i}$) is the lesser of the raster-scale soil retention supply and demand.  $\begin{aligned} {AS}_{SR,i}={AD}_{SR,i}=min({PS}_{SR,i},{PD}_{SR,i}) \end{aligned}$  **Note:** County scale actual supply calculations ${AS}_{k,j}$ serve only as an intermediate step for deriving the realization rate $\rho_{j}$ and the input-output analysis between counties; all final supply and demand results are presented and analyzed at the raster scale. |
| VP |  |  |  |  |
| MP |  |  |  |  |
| WY | Based on the ARIES model (Baró et al., 2016):  $\begin{aligned} {PS}_{WY,i}=(1-\frac{{AET}_{i}}{P_{i}})\times P_{i} \end{aligned}$  Where $P_{i}$ is annual precipitation and ${AET}_{i}$ is the actual evapotranspiration at pixel *i*. | Based on provincial agricultural, industrial, domestic, and ecological water demand:  $\begin{aligned} {PD}_{WY,i}={AW}_{sum}\times\frac{{AGDP}_{i}}{{AGDP}_{sum}}+{IW}_{sum}\times\frac{{IGDP}_{i}}{{IGDP}_{sum}} \\ +{DW}_{sum}\times\frac{{POP}_{i}}{{POP}_{sum}}+{EW}_{sum}\times\frac{{NPP}_{i}}{{NPP}_{sum}} \end{aligned}$  Where ${AW}_{sum}$, ${IW}_{sum}$, ${DW}_{sum}$, and ${EW}_{sum}$ are the province-level agricultural, industrial, domestic, and ecological water consumption, respectively; ${AGDP}_{i}$, ${IGDP}_{i}$, ${POP}_{i}$, and ${NPP}_{i}$ are the pixel-level agricultural GDP, industrial GDP, population density, and NPP; ${AGDP}_{sum}$, ${IGDP}_{sum}$, ${POP}_{sum}$, and ${NPP}_{sum}$ are the national agricultural GDP, industrial GDP, population density, and NPP. | Based on by land use data and population raster data for provincial (agricultural, industrial, residential, and ecological) water use (X. Zhang et al., 2022).  $\begin{aligned} {AD}_{WY,i}=\frac{{{AW}_{sum}\times AL}_{i}}{{AL}_{sum}}+\frac{{IW}_{sum}\times{IL}_{i}}{{IL}_{sum}} \\ +\frac{{EW}_{sum}\times{EL}_{i}}{{EL}_{sum}}+\frac{{DW}_{sum}\times{POP}_{i}}{{POP}_{sum}} \end{aligned}$  Where ${AL}_{i}$, ${IL}_{i}$, ${EL}_{i}$ are the pixel’s agricultural, industrial, and ecological land use areas; ${AL}_{sum}$ ${IL}_{sum}$, ${EL}_{sum}$ are the total area of provincial’s agricultural, industrial, and ecological land. |  |
| SR | Based on RUSLE equation (Ouyang et al., 2020):  $\begin{aligned} {PS}_{SR,i}=R_{i}\times K_{i}\times L_{i}\times S_{i}\times(1-C_{i}\times P_{i}) \end{aligned}$  Where $R_{i}$ is the rainfall erosivity, $K_{i}$ is soil erosivity. $L_{i}$ and $S_{i}$ are slope length and gradient factor, respectively. $C_{i}$ is vegetation cover factor, which can be calculated by NDVI. $P_{i}$ is management measures factor, which can be calculated by slope (X. Wang et al., 2020). | Based on RUSLE equation, the potential demand of soil retention can be interpreted as the target soil retention amount that should be maintained (Ouyang et al., 2020) :  $\begin{aligned} {PD}_{SR,i}=R_{i}\times K_{i}\times L_{i}\times S_{i}\times C_{i}\times P_{i} \end{aligned}$ | The actual demand of soil retention is the lesser of the raster-scale soil retention potential supply and demand.  $\begin{aligned} {AS}_{SR,i}=min({PS}_{SR,i},{PD}_{SR,i}) \end{aligned}$ |  |
| CS | The potential supply of carbon sequestration can be approximated as a function of NPP (J. Wang et al., 2019):  $\begin{aligned} {PS}_{CS,i}=1.63\times{NPP}_{i} \end{aligned}$ | Based on carbon emission quota measurement (Table S2), carbon sequestration demand at the raster scale can be determined by the share of carbon emissions of the pixel (Kong et al., 2019; Tian & LIN, 2021).  $\begin{aligned} {PD}_{CS,i}={CE}_{sum}\times\frac{{CEQ}_{i}}{{CEQ}_{sum}} \end{aligned}$  Where ${CEQ}_{i}$ is the carbon emission quota of the pixel i, ${CEQ}_{sum}$ is the national carbon emission quota, ${CE}_{sum}$ is the national carbon emission. | Based on the product of total actual demand (the lesser of total potential supply and demand) and the raster’s share of the nationwide carbon emissions.  $\begin{aligned} {AD}_{CS,i}=\frac{{CE}_{i}}{{CE}_{sum}}\times\min\left( {CE}_{sum},{PS}_{CS,sum} \right) \end{aligned}$  Where ${CE}_{i}$ is the carbon emission at the raster scale. |  |
| AP | Based on the i-Tree model, calculated by the land use type (Baró et al., 2015):  $\begin{aligned} {PS}_{AP,i}=f({landuse}_{i}) \end{aligned}$  where $f({landuse}_{i})$ is the i-Tree–derived function mapping land‐use type at pixel *i* to annual pollutant removal capacity (Table S3). | Based on the air pollutant concentrations:  $\begin{aligned} {PD}_{AP,i}=\left\{ \begin{aligned} \left( \rho_{{PM}_{2.5},i}-35 \right)\times H\times A,\rho_{{PM}_{2.5}}>35 \\ 0,\rho_{{PM}_{2.5}}\leq35 \end{aligned} \right. \end{aligned}$  Where $\rho_{{PM}_{2.5}}$ is the PM_2.5_ concentration at the pixel scale; H is the range of height of air purification, generally taken as 200m (J. Chen et al., 2019); A is the pixel area (1km^2^). | Based on the product of the actual demand (the lesser of total potential supply and demand) and the raster’s share of the nationwide air purification potential demand.  $\begin{aligned} {AD}_{AP,i}=\frac{{PD}_{AP,i}}{{PD}_{AP,sum}}\times\min\left( {PS}_{AP,sum},{PD}_{AP,sum} \right) \end{aligned}$ |  |
| HQ | Calculated in InVEST model (Hu et al., 2023; Sharp et al., 2015).  $\begin{aligned} {PS}_{HQ,i}=H_{j}\times\left[ 1-\left( \frac{D_{ij}^{Z}}{D_{ij}^{Z}+K^{Z}} \right) \right] \end{aligned}$  Where $H_{j}$ is the habitat suitability for habitat type *j*, $D_{ij}$ is the degree of habitat degradation in pixel *i* belonging to habitat type j (Table S4), K is the half-saturation constant, which is 0.05 in default, Z is the default parameter for the normalized constant model, which is 2.5 in this study. | Based on the human footprint index (HFI) and the proportion of the raster HFI to the national HFI (An et al., 2024).  $\begin{aligned} {HFI}_{i}=\frac{{LULC}_{i}+{POP}_{i}+{NLI}_{i}+{CE}_{i}+{GDP}_{i}}{5} \end{aligned}$  Where ${LULC}_{i}$, ${POP}_{i}$, ${NLI}_{i}$, ${CE}_{i}$, and ${GDP}_{i}$ are the land use intensity, population density, nighttime light intensity, carbon emission and GDP of the normalized pixel *i*, respectively.  $\begin{aligned} {PD}_{HQ,i}=\frac{{HFI}_{i}}{{HFI}_{x_{sum}}}\times{PS}_{HQ,sum} \end{aligned}$  Where ${HFI}_{sum}$, ${PS}_{HQ,sum}$ are the total HFI, habitat quality potential supply, respectively. | According to this study’s definition, the nationwide habitat quality and tourism recreation had an equivalent supply and demand. Therefore, the raster scale habitat quality and tourism recreation potential demands can be fully met.  $\begin{aligned} {AD}_{HQ,i}={PD}_{HQ,i} \end{aligned}$  $\begin{aligned} {AD}_{TR,i}={PD}_{TR,i} \end{aligned}$ |  |
| TR | Based on the weight of this raster in the kernel density of the city’s tourism value (Y. Zhang & Wang, 2025).  $\begin{aligned} {PS}_{TR,i}={TR}_{sum}\times\frac{k_{i}}{k_{sum}} \end{aligned}$  Where ${TR}_{sum}$ are the tourism revenue of the city; $k_{i}$ and $k_{sum}$ are the kernel density value of pixel i and the city, respectively (Table S5). | Based on the ecosystem service demand function and the proportion of the raster ecosystem service demand to the national demand (Liu et al., 2023).  $\begin{aligned} X_{TR,i}^{*}={LULC}_{i}\times\ln\left( {POP}_{i} \right)\times\ln\left( {GDP}_{i} \right) \end{aligned}$  Where $X_{TR}^{*}$ is the weight of tourism recreation demand.  $\begin{aligned} {PD}_{TR,i}=\frac{X_{TR,i}^{*}}{X_{TR_{sum}}^{*}}\times{TR}_{sum} \end{aligned}$  Where $X_{TR_{sum}}^{*}$ is the sum of tourism recreation demand weights nationwide. |  |  |

**Note:** For HQ, demand is defined as the societal requirement for biodiversity security and ecological integrity (Terrado et al., 2016). HQ is therefore treated as a state-based service, and its actual supply equals its estimated potential supply (AS = PS). For TR, demand represents the public desire and potential intensity of nature-based recreation (Vallecillo et al., 2019). The PS indicator is operationalized using an activity-based observational proxy and treated as actual supply in the ESF accounting framework (AS = PS).

**Section Ⅱ Biophysical and socio-economic parameters for ES assessment**

**Table S2.** Carbon sequestration demand measurement Indicators

| Principle | Indicator | Attribute |
| --- | --- | --- |
| Equality | GDP | ＋ |
|  | Population density | ＋ |
| Efficiency | Carbon emission intensity | － |
| Security | Carbon sink | ＋ |

**Table S3**. Average PM_2.5_ purification capacity for different land use types(t/km²)

| Land use type | Farmland | Forest | Grassland | Water body | Built-up land | Unused land |
| --- | --- | --- | --- | --- | --- | --- |
| Capture capacity | 0.25 | 2 | 1.07 | 0.18 | 0 | 0 |

**Table S4.** Parameters for habitat quality

|  | Threats |  |  |  |  |
| --- | --- | --- | --- | --- | --- |
|  |  | HABITAT | Farmland | Construction land | Unused land |
| The properties of threats | MAX_DIST (km) | - | 5.0 | 8.0 | 3.0 |
|  | WEIGHT | - | 0.6 | 1.0 | 0.2 |
|  | DECAY | - | linear | exponential | exponential |
| Sensitivity of different land use types | Farmland | 0.30 | 0.30 | 0.50 | 0.40 |
|  | Forest | 1.00 | 0.60 | 0.80 | 0.50 |
|  | Grassland | 0.70 | 0.50 | 0.40 | 0.50 |
|  | Water body | 0.80 | 0.70 | 0.60 | 0.20 |
|  | Built-up land | 0.00 | 0.00 | 0.00 | 0.00 |
|  | Unused land | 0.00 | 0.00 | 0.00 | 0.00 |

**Table S5.** Weights assigned to tourism-spot revenue levels

| Level | Tourism spot number | Total tourism revenue  (billion CNY) | Average tourism spot  revenue (million CNY ) | Weight |
| --- | --- | --- | --- | --- |
| 5A | 280 | 213.21 | 761.46 | 164.42 |
| 4A | 3720 | 215.99 | 58.06 | 12.54 |
| 3A | 6198 | 65.59 | 10.58 | 2.28 |
| 2A | 2101 | 11.37 | 5.41 | 1.17 |
| 1A | 103 | 0.48 | 4.66 | 1 |

Data sources: Ministry of Culture and Tourism, PRC

**Section Ⅲ Consistency and plausibility assessment**

**Figure S1**. Cross-validation of provisioning service supply


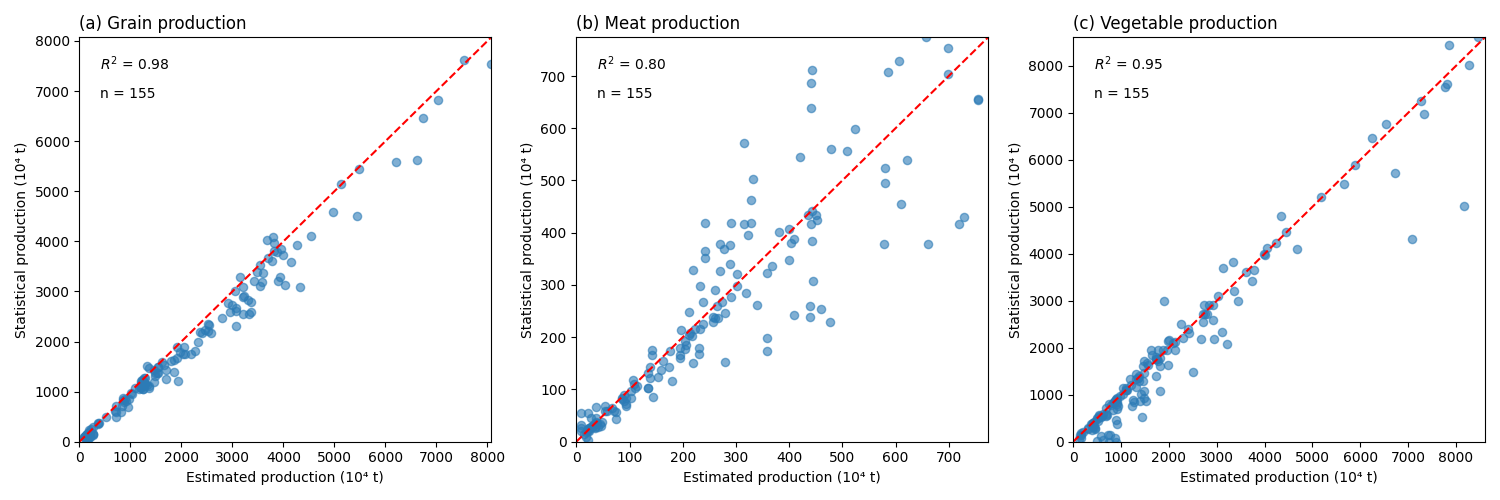


**Note:** Each point represents a province–year observation aggregated from county-level data (n = 31 provinces × 5 years =155). The dashed line indicates the 1:1 reference line. The coefficient of determination (R²) is reported for each panel to indicate the consistency between the estimated provisioning service supply and official statistical records. Validation statistical data were derived from National Bureau of Statistics of China (https://data.stats.gov.cn/english/easyquery.htm?cn=E0103)

**Table S6**. Spatial validation of PS patterns through comparison with previous studies

| **Indicator** | **Main spatial pattern in this study** | **Pattern reported in previous studies** | **Agreement level** | **References** |
| --- | --- | --- | --- | --- |
| GP, MP, VP | GP, VP concentrated in the North China Plain, Middle-Lower Yangtze Plain, and Sichuan Basin; MP concentrated in Agro-pastoral ecotone in northern China and southwest provinces (Yunnan and Guizhou) | High provisioning product supply was distributed in the region to the eastern Hu Huanyong Line | High agreement | Luo et al. 2026 |
|  |  | High GP was reported in the Central China Region and East China Region. | Moderate agreement | Zhang et al., 2025 |
|  |  | High values of provisioning product were concentrated in the middle and lower reaches of the Yangtze River Plain, North China Plain, Northeast Plain, and Sichuan Basin, whereas low areas were mainly observed in northwestern China. | High agreement | Gao et al., 2025 |
|  |  | Cities with the highest material supply were concentrated primarily in the eastern and southern regions. | Moderate agreement | Liu et al., 2025 |
| WY | Mainly distributed in mountainous areas south of the Yangtze River, including the Wuyi, Nanling, and Wuling Mountains, and also in the Changbai Mountains and the Greater and Lesser Khingan Ranges in Northeast China. | WY was higher in southern China and lower in northern China. | High agreement | Luo et al., 2026 |
|  |  | WY exhibited a gradient distribution that decreased from the northwestern inland to the southeastern coastal, coinciding with the Heihe–Tengchong line. | Partial agreement | Gao et al., 2025 |
|  |  | WY was concentrated in southwestern and northeastern cities. | High agreement | Liu et al., 2025 |
|  |  | Southern and southeastern coastal cities recorded higher WY levels, while northwestern arid cities had lower values. | High agreement | Su et al., 2024 |
|  |  | The South China Region produces the highest WY, primarily in northern and southeastern coastal areas. | Moderate agreement | Zhang et al., 2025 |
|  |  | WY is higher in the southeast and lower in the northwest, gradually decreasing from east to west. | High agreement | Liu et al., 2026 |
| SR | High values were primarily distributed in the Qinghai–Tibet Plateau, Loess Plateau, and Qinba Mountains. | High-value areas for SR showed fragmentation and relative concentration in the southwestern region, while SC supply capacity was generally low north of the Qinling Mountains and in the southwest karst areas. | Moderate agreement | Gao et al., 2025 |
|  |  | SR service hotspots were found in Linzhi, Tibet, and Chongqing. | Moderate agreement | Liu et al., 2025 |
|  |  | SR service levels were high in plateau and mountainous areas such as the Tibetan Plateau. | High agreement | Su et al., 2024 |
|  |  | The East China Region and South China Region have the highest SR. | Partial agreement | Zhang et al., 2025 |
|  |  | SR is primarily concentrated in the Sichuan Basin, Loess Plateau, surrounding mountainous and hilly areas, eastern Great Khingan, and the Yunnan–Guizhou Plateau. | Moderate agreement | Liu et al., 2026 |
| CS | Prominently distributed in forest-rich regions such as the Yunnan–Guizhou Plateau and Changbai Mountains, whereas desertification regions in northwestern China recorded the lowest supply. | CS exhibited a decreasing trend from the southeast to the northwest. Higher CS was primarily concentrated in Yunnan, the southern Shaanxi Qinling Mountains, the Daxing’anling and Xiaoxing’anling Mountains in Northeast China, and the southeastern coastal areas. | High agreement | Luo et al., 2026 |
|  |  | High-supply areas were predominantly distributed in the Yunnan–Guizhou Plateau, Southeast Hills, the Small Khingan Mountains, Changbai Mountains, and the Middle and Lower Yangtze River Plain, while low areas were predominantly concentrated in the North Plateau region and the northwest. | High agreement | Gao et al., 2025 |
|  |  | CS was relatively high in Inner Mongolia, especially in Xilin Gol League and Hulunbuir City. | Partial agreement | Liu et al., 2025 |
|  |  | The South China Region provides the highest CS. | Moderate agreement | Zhang et al., 2025 |
| AP | Stronger capacity occurred in southern regions and northeastern forested areas. | Significant values were observed in cities such as Qiqihar in Northeast China and Chongqing in Southwest China. | Moderate agreement | Liu et al., 2025 |
| HQ | High-quality areas were primarily distributed in the Qinghai–Tibet Plateau and mountainous regions, whereas low-quality areas occurred in desertified northwestern regions. | High-supply areas were predominantly distributed in the Yunnan–Guizhou Plateau, Southeast Hills, the Small Khingan Mountains, Changbai Mountains, and the Middle and Lower Yangtze River Plain, while low areas were predominantly concentrated in the North Plateau region and the northwest. | Moderate agreement | Gao et al., 2025 |
|  |  | HQ declined in eastern coastal and central regions, while western regions saw modest improvements. | High agreement | Su et al., 2024 |
|  |  | The Southwest China Region has the highest HQ. | High agreement | Zhang et al., 2025 |
| TR | Tourism recreation supply evolved from scattered to continuous spatial distribution in the southeastern areas. | Southeast and Northeast China were the high-supply areas, whereas the North China Plain occupied a low position. | Moderate agreement | Wu et al., 2019 |
|  |  | Cities with the highest TR values were concentrated primarily in the southeastern and southern regions, such as Hangzhou and Guangzhou. | High agreement | Liu et al., 2025 |
|  |  | The Central China Region, East China Region, and South China Region demonstrate strong TR capacities. | High agreement | Zhang et al., 2025 |

Note: This table summarizes the spatial patterns by comparing the spatial distributions identified in this study (PS) with those reported in previous studies (ES supply). Agreement levels (high, moderate, and partial) were determined based on the overall consistency of major spatial patterns rather than exact geographic matches. High agreement indicates that the dominant spatial distribution patterns are consistent with those reported in previous studies; moderate agreement indicates broadly similar patterns with some regional differences; and partial agreement indicates limited consistency. Descriptions of spatial patterns from previous studies were summarized and harmonized to facilitate comparison.

**Table S7.** Spatial validation of PD patterns through comparison with previous studies

| **Indicator** | **Main spatial pattern in this study** | **Pattern reported in previous studies** | **Agreement level** | **References** |
| --- | --- | --- | --- | --- |
| GP, MP, VP | Potential grain, meat, and vegetable demand were concentrated in densely populated urban clusters, particularly in southeastern China. | Provisioning product demand showed a spatial pattern that was higher in the east and lower in the west. | High agreement | Luo et al., 2026 |
|  |  | Demand centers were Beijing, the Pearl River Delta, and the Yangtze River Delta. | High agreement | Wu et al., 2019 |
|  |  | Areas with high demand were primarily concentrated in economically active areas such as the Beijing–Tianjin–Hebei region, Yangtze River Delta, Chengdu–Chongqing economic circle, Guangdong–Hong Kong–Macao Greater Bay Area, and the eastern coastal areas. | High agreement | Gao et al., 2025 |
|  |  | High demand occurred in high population-density areas. | High agreement | Zhang et al., 2025 |
| WY | Potential water-yield demand was primarily distributed in densely populated and agriculturally productive areas such as the North China Plain, Northeast China Plain, and rapidly urbanizing areas such as the Chengdu–Chongqing region. | WY demand showed an overall downward trend from southeast to northwest, closely aligning with population density and industrial distribution patterns. | Moderate agreement | Luo et al., 2026 |
|  |  | Areas with high demand were primarily concentrated in economically active areas such as the Beijing–Tianjin–Hebei region, Yangtze River Delta, Chengdu–Chongqing economic circle, Guangdong–Hong Kong–Macao Greater Bay Area, and the eastern coastal areas. | Moderate agreement | Gao et al., 2025 |
|  |  | WY increased primarily in North China, East China, and the Pearl River Delta, while Central and Northwest China saw a slight decrease. | High agreement | Su et al., 2024 |
|  |  | High demand occurred in East China Region. | Moderate agreement | Zhang et al., 2025 |
| SR | Potential soil-retention demand was particularly high in desertified and erosion-prone areas in northwestern China. | High values of SR demand were predominantly found in the Yellow River Basin, the Yunnan–Guizhou Plateau, and southern hilly areas. | Moderate agreement | Gao et al., 2025 |
|  |  | High levels occurred in Eastern and Southern China, especially in hilly areas, with decreases in Southwest and Northwest China. | Partial agreement | Su et al., 2024 |
|  |  | South China Region and Southwest China Region exhibit high SR demand. | Partial agreement | Zhang et al., 2025 |
| CS | Potential carbon-sequestration demand was mainly distributed in southwest and northeast regions in response to rising carbon emissions. | High-value areas were mainly concentrated in the North China Plain, Sichuan Basin, Yangtze River Delta, and Pearl River Delta, while low-value areas were found in Xinjiang, Tibet, Qinghai, western Sichuan, and Yunnan. | Partial agreement | Luo et al., 2026 |
|  |  | Areas with high demand were primarily concentrated in economically active areas such as the Beijing–Tianjin–Hebei region, Yangtze River Delta, Chengdu–Chongqing economic circle, Guangdong–Hong Kong–Macao Greater Bay Area, and the eastern coastal areas. | Partial agreement | Gao et al., 2025 |
|  |  | High demand occurred in high population-density areas. | Partial agreement | Zhang et al., 2025 |
| AP | Potential air-purification demand was predominantly concentrated in the North China Plain and southern Xinjiang. | No directly matched AP demand study was identified among the collected references. | — | — |
| HQ | Potential habitat-quality demand was higher in economically active regions such as the North China Plain and Yangtze River Delta. | Areas with high demand were primarily concentrated in economically active areas such as the Beijing–Tianjin–Hebei region, Yangtze River Delta, Chengdu–Chongqing economic circle, Guangdong–Hong Kong–Macao Greater Bay Area, and the eastern coastal areas. | High agreement | Gao et al., 2025 |
|  |  | High levels occurred in eastern coastal cities and increased in Guangdong province, while cities in the Tibetan Plateau had lower demand. | High agreement | Su et al., 2024 |
|  |  | HQ demand high areas are centralized in megacities and deserts of northwestern China. | Moderate agreement | Zhang et al., 2025 |
| TR | Potential tourism-recreation demand expanded from initially scattered urban centers to extensive southeastern regions. | Demand centers were Beijing, the Pearl River Delta, and the Yangtze River Delta. | High agreement | Wu et al., 2019 |
|  |  | High demand occurred in high population-density areas. | High agreement | Zhang et al., 2025 |

Note: This table summarizes the spatial patterns by comparing the spatial distributions identified in this study (PD) with those reported in previous studies (ES demand). Agreement levels (high, moderate, and partial) were determined based on the overall consistency of major spatial patterns rather than exact geographic matches. High agreement indicates that the dominant spatial distribution patterns are consistent with those reported in previous studies; moderate agreement indicates broadly similar patterns with some regional differences; and partial agreement indicates limited consistency. Descriptions of spatial patterns from previous studies were summarized and harmonized to facilitate comparison.

**Section Ⅳ Algorithmic verification**

**Table S8.** Algorithmic validation summary for the AS and AD allocation

| Validation item |  | Indicator | 2000 | 2005 | 2010 | 2015 | 2020 |
| --- | --- | --- | --- | --- | --- | --- | --- |
| Non-negativity of inter-county transfer matrix entries |  | — | 0 | 0 | 0 | 0 | 0 |
| No simultaneous non-zero inflow and outflow for the same county |  | — | 0 | 0 | 0 | 0 | 0 |
| National closure | AS | GP (10^8^ t) | 2.52 | 2.15 | 1.93 | 1.83 | 1.89 |
|  |  | MP (10^8^ t) | 0.28 | 0.32 | 0.38 | 0.48 | 0.53 |
|  |  | VP (10^8^ t) | 1.32 | 1.32 | 1.32 | 1.35 | 1.44 |
|  |  | WY (10^11^ m^3^) | 5.43 | 5.53 | 5.91 | 5.99 | 5.54 |
|  |  | SR (10^9^ t) | 8.22 | 9.21 | 8.62 | 8.52 | 8.74 |
|  |  | CS (10^9^ t) | 0.62 | 1.07 | 1.57 | 1.82 | 2.08 |
|  |  | AP (10^10^ t) | 8.18 | 8.17 | 8.17 | 8.15 | 7.89 |
|  |  | HQ (10^6^ km²·index) | 1.31 | 1.40 | 1.47 | 1.56 | 1.60 |
|  |  | TR (10^12^ CNY) | 0.90 | 1.99 | 4.73 | 11.25 | 23.05 |
|  | AD | GP (10^8^ t) | 2.52 | 2.15 | 1.93 | 1.83 | 1.89 |
|  |  | MP (10^8^ t) | 0.28 | 0.32 | 0.38 | 0.48 | 0.53 |
|  |  | VP (10^8^ t) | 1.32 | 1.32 | 1.32 | 1.35 | 1.44 |
|  |  | WY (10^11^ m^3^) | 5.43 | 5.53 | 5.91 | 5.99 | 5.54 |
|  |  | SR (10^9^ t) | 8.22 | 9.21 | 8.62 | 8.52 | 8.74 |
|  |  | CS (10^9^ t) | 0.62 | 1.07 | 1.57 | 1.82 | 2.08 |
|  |  | AP (10^10^ t) | 8.18 | 8.17 | 8.17 | 8.15 | 7.89 |
|  |  | HQ (10^6^ km²·index) | 1.31 | 1.40 | 1.47 | 1.56 | 1.60 |
|  |  | TR (10^12^ CNY) | 0.90 | 1.99 | 4.73 | 11.25 | 23.05 |

Note: This table summarizes the algorithmic validation of the IPF-based inter-county allocation of ecosystem services. “Non-negativity of inter-county transfer matrix entries” reports the minimum value of the balanced transfer matrix for each year; values of 0 indicate that no negative transfers were generated during the balancing procedure. “No simultaneous non-zero inflow and outflow for the same county” reports the number of counties that simultaneously had non-zero inflow and outflow in the same indicator-year matrix; values of 0 indicate that no such logical conflict occurred. “National closure” verifies that the national totals of actual supply (AS) and actual demand (AD) are identical for each indicator and year. Units for each ecosystem service are indicated in the corresponding indicator labels.

**Table S9.** Algorithmic validation summary for ESF decomposition

| Validation item |  | Indicator | 2000 | 2005 | 2010 | 2015 | 2020 |
| --- | --- | --- | --- | --- | --- | --- | --- |
| ESF decomposition identity | ESF | GP (10^8^ t) | 2.52 | 2.15 | 1.93 | 1.83 | 1.89 |
|  |  | MP (10^8^ t) | 0.28 | 0.32 | 0.38 | 0.48 | 0.53 |
|  |  | VP (10^8^ t) | 1.32 | 1.32 | 1.32 | 1.35 | 1.44 |
|  |  | WY (10^11^ m^3^) | 5.43 | 5.53 | 5.90 | 5.99 | 5.54 |
|  |  | CS (10^9^ t) | 0.62 | 1.07 | 1.57 | 1.82 | 2.08 |
|  |  | AP (10^10^ t) | 8.18 | 8.17 | 8.17 | 8.15 | 7.89 |
|  |  | HQ (10^6^ km²·index) | 1.31 | 1.39 | 1.47 | 1.56 | 1.60 |
|  |  | TR (10^12^ CNY) | 0.90 | 1.99 | 4.73 | 11.25 | 23.05 |
|  | ISF | GP (10^8^ t) | 1.21 | 1.03 | 0.85 | 0.78 | 0.76 |
|  |  | MP (10^8^ t) | 0.03 | 0.03 | 0.03 | 0.04 | 0.04 |
|  |  | VP (10^8^ t) | 0.65 | 0.66 | 0.60 | 0.60 | 0.59 |
|  |  | WY (10^11^ m^3^) | 0.87 | 0.98 | 1.06 | 1.12 | 1.06 |
|  |  | CS (10^9^ t) | 0.27 | 0.35 | 0.44 | 0.47 | 0.54 |
|  |  | AP (10^10^ t) | 2.98 | 4.17 | 4.19 | 3.29 | 0.47 |
|  |  | HQ (10^6^ km²·index) | 0.45 | 0.45 | 0.46 | 0.45 | 0.42 |
|  |  | TR (10^12^ CNY) | 0.12 | 0.28 | 0.75 | 1.91 | 4.13 |
|  | IF | GP (10^8^ t) | 1.13 | 1.00 | 0.91 | 0.86 | 0.82 |
|  |  | MP (10^8^ t) | 0.18 | 0.23 | 0.24 | 0.29 | 0.29 |
|  |  | VP (10^8^ t) | 0.57 | 0.60 | 0.58 | 0.60 | 0.64 |
|  |  | WY (10^11^ m^3^) | 1.61 | 1.61 | 1.68 | 1.72 | 1.72 |
|  |  | CS (10^9^ t) | 0.16 | 0.27 | 0.35 | 0.36 | 0.46 |
|  |  | AP (10^10^ t) | 0.60 | 0.61 | 0.64 | 0.61 | 0.31 |
|  |  | HQ (10^6^ km²·index) | 0.25 | 0.25 | 0.26 | 0.27 | 0.28 |
|  |  | TR (10^12^ CNY) | 0.17 | 0.41 | 1.18 | 3.22 | 7.25 |
|  | EF | GP (10^8^ t) | 0.18 | 0.12 | 0.17 | 0.19 | 0.31 |
|  |  | MP (10^8^ t) | 0.07 | 0.06 | 0.11 | 0.15 | 0.20 |
|  |  | VP (10^8^ t) | 0.10 | 0.06 | 0.14 | 0.15 | 0.21 |
|  |  | WY (10^11^ m^3^) | 2.95 | 2.94 | 3.16 | 3.15 | 2.76 |
|  |  | CS (10^9^ t) | 0.18 | 0.45 | 0.78 | 0.99 | 1.08 |
|  |  | AP (10^10^ t) | 4.60 | 3.39 | 3.35 | 4.25 | 7.11 |
|  |  | HQ (10^6^ km²·index) | 0.61 | 0.69 | 0.75 | 0.84 | 0.90 |
|  |  | TR (10^12^ CNY) | 0.61 | 1.30 | 2.80 | 6.12 | 11.67 |
| EF inconsistency with the transfer matrix |  | — | 0 | 0 | 0 | 0 | 0 |

Note: This table reports the validation of ecosystem service flow (ESF) decomposition consistency. According to the accounting framework, ESF is decomposed into in-situ flow (ISF), interior flow (IF), and exterior flow (EF), therefore ESF = ISF + IF + EF. The table presents the corresponding values for each indicator and year to verify this identity. EF represents the cross-county flow component derived from the balanced transfer matrix, while ISF and IF represent the locally realized and internally allocated portions of ecosystem service flows, respectively. “EF inconsistency with the transfer matrix” reports the residual between EF and the corresponding cross-county transfer totals; values of **0** indicate that EF is fully consistent with the transfer matrix and that no discrepancy exists between the ESF decomposition and the allocation results. Units are shown in the indicator labels.

**Section Ⅴ Robustness and sensitivity analysis**

**Table S10.** Scenario setting for sensitive analysis

| **Scenario** | **Purpose** | **Included ES indicators** | **Zoning threshold** |
| --- | --- | --- | --- |
| S0 | Baseline | 8 indicators | ±0.10 |
| S1 | Threshold sensitivity | 8 indicators | ±0.05 |
| S2 | Threshold sensitivity | 8 indicators | ±0.15 |
| S3 | ES inclusion sensitivity | 7 indicators (excluding HQ) | ±0.10 |
| S4 | ES inclusion sensitivity | 7 indicators (excluding TR) | ±0.10 |
| S5 | ES inclusion sensitivity | 6 indicators (excluding HQ and TR) | ±0.10 |

**Table S11.** County number of zoning types and largest class share across scenarios

| Scenario | Year | Local Sustained | Local Pressured | Dynamic Transitional | External Sustained | External Pressured | Largest Class Share |
| --- | --- | --- | --- | --- | --- | --- | --- |
| S0 | 2000 | 912 | 302 | 758 | 838 | 36 | 0.320 |
|  | 2005 | 773 | 472 | 694 | 835 | 72 | 0.293 |
|  | 2010 | 633 | 628 | 630 | 862 | 93 | 0.303 |
|  | 2015 | 704 | 498 | 646 | 927 | 71 | 0.326 |
|  | 2020 | 721 | 381 | 767 | 954 | 23 | 0.335 |
| S1 | 2000 | 1058 | 353 | 373 | 1006 | 56 | 0.372 |
|  | 2005 | 921 | 528 | 314 | 990 | 93 | 0.348 |
|  | 2010 | 740 | 693 | 305 | 979 | 129 | 0.344 |
|  | 2015 | 809 | 567 | 324 | 1044 | 102 | 0.367 |
|  | 2020 | 922 | 423 | 383 | 1087 | 31 | 0.382 |
| S2 | 2000 | 695 | 250 | 1209 | 669 | 23 | 0.425 |
|  | 2005 | 623 | 386 | 1094 | 693 | 50 | 0.384 |
|  | 2010 | 516 | 541 | 986 | 738 | 65 | 0.346 |
|  | 2015 | 552 | 428 | 995 | 821 | 50 | 0.350 |
|  | 2020 | 488 | 321 | 1211 | 813 | 13 | 0.426 |
| S3 | 2000 | 1186 | 339 | 710 | 577 | 34 | 0.417 |
|  | 2005 | 1035 | 525 | 648 | 583 | 55 | 0.364 |
|  | 2010 | 845 | 719 | 579 | 640 | 63 | 0.297 |
|  | 2015 | 937 | 588 | 562 | 708 | 51 | 0.329 |
|  | 2020 | 1097 | 404 | 608 | 706 | 31 | 0.385 |
| S4 | 2000 | 1107 | 391 | 641 | 685 | 22 | 0.389 |
|  | 2005 | 928 | 571 | 580 | 720 | 47 | 0.326 |
|  | 2010 | 730 | 737 | 543 | 776 | 60 | 0.273 |
|  | 2015 | 806 | 607 | 518 | 864 | 51 | 0.304 |
|  | 2020 | 942 | 445 | 581 | 865 | 13 | 0.331 |
| S5 | 2000 | 1501 | 404 | 516 | 406 | 19 | 0.527 |
|  | 2005 | 1248 | 607 | 513 | 455 | 23 | 0.439 |
|  | 2010 | 981 | 825 | 476 | 542 | 22 | 0.345 |
|  | 2015 | 1060 | 667 | 463 | 636 | 20 | 0.372 |
|  | 2020 | 1280 | 452 | 465 | 640 | 9 | 0.450 |

**Table S12.** Temporal stability metrics of zoning outcomes across scenarios

| Scenario | Mean Dominant Share | Mean Transition Rate |
| --- | --- | --- |
| S0 | 0.850 | 0.193 |
| S1 | 0.855 | 0.189 |
| S2 | 0.857 | 0.186 |
| S3 | 0.842 | 0.200 |
| S4 | 0.862 | 0.175 |
| S5 | 0.849 | 0.187 |

**Table S13.** Structural similarity between alternative scenarios and the baseline zoning

|  | S1 | S2 | S3 | S4 | S5 |
| --- | --- | --- | --- | --- | --- |
| 2000 | 0.865 | 0.842 | 0.738 | 0.795 | 0.616 |
| 2005 | 0.866 | 0.859 | 0.747 | 0.812 | 0.651 |
| 2010 | 0.886 | 0.875 | 0.771 | 0.835 | 0.682 |
| 2015 | 0.887 | 0.877 | 0.772 | 0.836 | 0.699 |
| 2020 | 0.865 | 0.844 | 0.718 | 0.804 | 0.659 |
| Average | 0.874 | 0.859 | 0.749 | 0.816 | 0.661 |

**References**

1. An, Q., Yuan, X., Zhang, X., Yang, Y., Chen, J., & An, J. (2024). Spatio-temporal interaction and constraint effects between ecosystem services and human activity intensity in Shaanxi Province, China. *Ecological Indicators, 160*, 111937.
2. Baró, F., Haase, D., Gómez-Baggethun, E., & Frantzeskaki, N. (2015). Mismatches between ecosystem services supply and demand in urban areas: A quantitative assessment in five European cities. *Ecological Indicators, 55*, 146-158.
3. Baró, F., Palomo, I., Zulian, G., Vizcaino, P., Haase, D., & Gómez-Baggethun, E. (2016). Mapping ecosystem service capacity, flow and demand for landscape and urban planning: A case study in the Barcelona metropolitan region. *Land Use Policy, 57*, 405-417.
4. Chen, J., Jiang, B., Bai, Y., Xu, X., & Alatalo, J. M. (2019). Quantifying ecosystem services supply and demand shortfalls and mismatches for management optimisation. *Science of The Total Environment, 650*, 1426-1439.
5. Chen, Q., Gao, Y., Pan, C., Xu, D., Cai, K., Guan, D., et al. (2023). An interprovincial input–output database distinguishing firm ownership in China from 1997 to 2017. *Scientific Data, 10*(1), 293.
6. Chinese Nutrition Society. (2022). *The Chinese Dietary Guidelines: 2022*. Beijing: People’s Medical Publishing House.
7. De Petrillo, E., Fahrländer, S. F., Tuninetti, M., Andersen, L. S., Monaco, L., Ridolfi, L., & Laio, F. (2025). Reconciling tracked atmospheric water flows to close the global freshwater cycle. *Communications Earth & Environment, 6*(1), 347.
8. Gao, M., Hu, Y., Liu, X., & Liang, M. (2025). Revealing multi-scale characteristics of ecosystem services supply and demand imbalance to enhance refined ecosystem management in China. *Ecological Indicators*, *170*, 112971.
9. Hu, B., Li, Z., Wu, H., Han, H., Cheng, X., & Kang, F. (2023). Coupling strength of human-natural systems mediates the response of ecosystem services to land use change. *Journal of Environmental Management, 344*, 118521.
10. Kong, Y., Zhao, T., Yuan, R., & Chen, C. (2019). Allocation of carbon emission quotas in Chinese provinces based on equality and efficiency principles. *Journal of Cleaner Production, 211*, 222-232.
11. Kuri, F., Murwira, A., Murwira, K. S., & Masocha, M. (2014). Predicting maize yield in Zimbabwe using dry dekads derived from remotely sensed Vegetation Condition Index. *International Journal of Applied Earth Observation and Geoinformation, 33*, 39-46.
12. Liu, Q., Liu, H., Xu, G., Lu, B., Wang, X., & Li, J. (2023). Spatial gradients of supply and demand of ecosystem services within cities. *Ecological Indicators, 157*, 111263.
13. Liu, Y., Zhao, W., Zhang, Z., Ding, J., & Wang, L. (2026). A 30 m spatial resolution dataset of ecosystem services in China for 2000, 2010, and 2020. *Earth System Science Data*, *18*(2), 1519-1540.
14. Liu, Zhaopu, et al. "Spatiotemporal dynamics of gross ecosystem product in Chinese cities: Pathways to sustainable urban development." *Journal of Cleaner Production* 519 (2025): 146033.
15. Luo, Q., Qi, Z., Zhou, J., Zeng, X., & Chu, Y. (2026). Dynamic and static matching of ecosystem service supply-demand for ecological management zoning: A water-energy-food perspective in China's county-level cities. *Journal of Cleaner Production*, *538*, 147293.
16. Ouyang, Z., Song, C., Zheng, H., Polasky, S., Xiao, Y., Bateman, I. J., et al. (2020). Using gross ecosystem product (GEP) to value nature in decision making. *Proceedings of the National academy of Sciences, 117*(25), 14593-14601.
17. Sharp, R., Tallis, H., Ricketts, T., Guerry, A., Wood, S., Chaplin-Kramer, R., et al. (2015). InVEST 3.2. 0 user’s guide. *The natural capital project*, 133.
18. Su, R., Duan, C., & Chen, B. (2024). The shift in the spatiotemporal relationship between supply and demand of ecosystem services and its drivers in China. *Journal of Environmental Management*, *365*, 121698.
19. Terrado, M., Sabater, S., Chaplin-Kramer, B., Mandle, L., Ziv, G., & Acuña, V. (2016). Model development for the assessment of terrestrial and aquatic habitat quality in conservation planning. *Science of the total environment*, *540*, 63-70.
20. Tian, Y., & LIN, Z. (2021). Provincial distribution of China's carbon emission rights and assessment of its emission reduction potential under the Paris Agreement. *Journal of Natural Resources, 36*(4), 921-933 (In Chinese).
21. Vallecillo, S., La Notte, A., Zulian, G., Ferrini, S., & Maes, J. (2019). Ecosystem services accounts: Valuing the actual flow of nature-based recreation from ecosystems to people. *Ecological Modelling*, *392*, 196-211.
22. Wang, J., Zhou, W., Pickett, S. T., Yu, W., & Li, W. (2019). A multiscale analysis of urbanization effects on ecosystem services supply in an urban megaregion. *Science of The Total Environment, 662*, 824-833.
23. Wang, X., Zhang, X., Feng, X., Liu, S., Yin, L., & Chen, Y. (2020). Trade-offs and synergies of ecosystem services in karst area of China driven by grain-for-green Program. *Chinese Geographical Science, 30*, 101-114.
24. Wu, X., Liu, S., Zhao, S., Hou, X., Xu, J., Dong, S., & Liu, G. (2019). Quantification and driving force analysis of ecosystem services supply, demand and balance in China. *Science of the Total Environment*, *652*, 1375-1386.
25. Zhang, J., Wang, M., Liu, K., Chen, S., & Zhao, Z. A. (2025). Social-ecological system sustainability in China from the perspective of supply-demand balance for ecosystem services. *Journal of Cleaner Production*, *497*, 145039.
26. Zhang, X., Wang, Y., Yuan, X., Shao, Y., & Bai, Y. (2022). Identifying ecosystem service supply-demand imbalance for sustainable land management in China’s Loess Plateau. *Land Use Policy, 123*, 106423.
27. Zhang, Y., & Wang, Y. (2025). How does urbanization evolve heterogeneously in urbanized, urbanizing, and rural areas of China? Insights from ecosystem service value. *Geography and Sustainability, 6*(3), 100254.
